# A Phosphoinositide-Binding Protein Acts in the Trafficking Pathway of Hemoglobin in the Malaria Parasite Plasmodium falciparum

**DOI:** 10.1128/mbio.03239-21

**Published:** 2022-01-18

**Authors:** Angana Mukherjee, Marie-Ève Crochetière, Audrey Sergerie, Souad Amiar, L. Alexa Thompson, Zeinab Ebrahimzadeh, Dominic Gagnon, Florian Lauruol, Alexandra Bourgeois, Thomas Galaup, Stéphanie Roucheray, Stéphanie Hallée, Prasad K. Padmanabhan, Robert V. Stahelin, Joel B. Dacks, Dave Richard

**Affiliations:** a Centre de Recherche en Infectiologie, CRCHU de Québec-Université Laval, Laurier Québec (QC), Canada; b Department of Microbiology-Infectious Diseases and Immunology, Faculty of Medicine, Laval University, Quebec City (Qc), Canada; c Department of Medicinal Chemistry and Molecular Pharmacology and the Purdue Institute of Inflammation, Immunology and Infectious Disease, Purdue University, West Lafayette, Indiana, USA; d Division of Infectious Disease, Department of Medicine, Faculty of Medicine and Dentistry, University of Alberta, Edmonton, Alberta, Canada; e Institute of Parasitology, Biology Center, CAS, Ceske Budejovice, Czech Republic; f Boler-Parseghian Center for Rare and Neglected Diseases, Department of Biological Sciences, University of Notre Dame, Notre Dame, Indiana, USA; University of Geneva

**Keywords:** hemoglobin, knockout, malaria, phosphoinositides, vacuoles, vesicular trafficking

## Abstract

Phosphoinositide lipids play key roles in a variety of processes in eukaryotic cells, but our understanding of their functions in the malaria parasite Plasmodium falciparum is still very much limited. To gain a deeper comprehension of the roles of phosphoinositides in this important pathogen, we attempted gene inactivation for 24 putative effectors of phosphoinositide metabolism. Our results reveal that 79% of the candidates are refractory to genetic deletion and are therefore potentially essential for parasite growth. Inactivation of the gene coding for a *Plasmodium*-specific putative phosphoinositide-binding protein, which we named PfPX1, results in a severe growth defect. We show that PfPX1 likely binds phosphatidylinositol-3-phosphate and that it localizes to the membrane of the digestive vacuole of the parasite and to vesicles filled with host cell cytosol and labeled with endocytic markers. Critically, we provide evidence that it is important in the trafficking pathway of hemoglobin from the host erythrocyte to the digestive vacuole. Finally, inactivation of PfPX1 renders parasites resistant to artemisinin, the frontline antimalarial drug. Globally, the minimal redundancy in the putative phosphoinositide proteins uncovered in our work supports that targeting this pathway has potential for antimalarial drug development. Moreover, our identification of a phosphoinositide-binding protein critical for the trafficking of hemoglobin provides key insight into this essential process.

## INTRODUCTION

The Plasmodium falciparum parasite is responsible for the most virulent form of malaria, a disease that is still taking a tremendous toll on humanity with over 241 million cases and 627,000 deaths in 2020. Over the past 20 years, important reductions in the number of malaria cases and deaths have been observed. Worryingly, however, this seems to have reached a plateau in recent years (1). The lack of a commercialized vaccine and the spread of resistance to the first line agent artemisinin (ART) in South East Asia demonstrate the urgency of finding new therapeutic targets (2–5).

Despite representing only 1% of the total phospholipid content of a eukaryotic cell (6), phosphoinositides (PPIs) play critical roles in a variety of processes such as cell motility, cytoskeletal reorganization, cell cycle and trafficking (7–9). PPIs are phosphorylated derivatives of phosphatidylinositol at positions 3, 4 and 5 that can be combined to generate seven species of PPIs through the action of lipid kinases and phosphatases (10). The various PPI species are not uniformly distributed throughout cellular membranes but instead are concentrated in specific organelles and membrane microdomains. For example, PI4P is prominently found at the Golgi apparatus (11) while PI3P is enriched in early endosomes (12). This PPI code recruits proteins containing domains with varying abilities to bind PPI species. Some of these possess exquisite binding specificity such as the Pleckstrin homology (PH) domain of PI-Phospholipase C delta for PI(4,5)P_2_ (13) and the phagocytic oxidase (PHOX, PX) domain of p40^phox^ for PI3P (14), while others are more relaxed in their binding specificity (15).

Recent reports have started to delve into the roles of PPIs in the lifecycle of P. falciparum (16). The parasite has the ability to generate numerous PPI species such as PI3P, PI4P and PI(4,5)P_2_ along with small amounts of PI(3,4)P_2_ and PI(3,4,5)P_3_ (17–19). Most of these have specific distributions throughout the erythrocytic cycle with phosphatidylinositol-3-phosphate (PI3P) found at the digestive vacuole (DV) membrane, host-cell cytosol-filled vesicles and the apicoplast, PI4P at the Golgi, and PI(4,5)P_2_ at the plasma membrane (17, 20, 21). Interestingly, the complement of PPI kinases and phosphatases of the parasite is quite restricted compared to other eukaryotes which could mean limited functional redundancy between the enzymes (16, 22). Importantly, the P. falciparum PI4KIIIb is a target of new classes of antimalarials currently in development (23, 24). The parasite’s PI-PLC homologue, through its degradation of PI(4,5)P_2_ into IP3 and diacylglycerol and the subsequent release of intracellular calcium stores, is important in numerous processes such as gametocyte activation (25–27), ookinete and sporozoite motility, and merozoite egress (28, 29). P. falciparum PPI-binding proteins have been implicated in processes such as merozoite invasion (30–32) and the cell cycle (33).

An important part of the parasite’s lifecycle is spent inside human erythrocytes which provide protection from the host’s immune system and also give it access to key nutrients such as hemoglobin (Hb). The digestion of Hb by proteases in the parasite’s DV, a lysosome-like organelle, is an important source of amino acids (34, 35). This process also generates free heme which the parasite converts to nontoxic hemozoin, a process targeted by antimalarial drugs such as chloroquine (36, 37). Hb trafficking to the DV is likely initiated at invaginations called cytostomes (38–41) and potentially proceeds to the DV membrane by vesicular transport (40, 42). Until recently, the molecular effectors involved in this process were unknown but it has now been demonstrated that the P. falciparum homologue of VPS45, a conserved protein involved in endolysosomal transport in other eukaryotes (43), was critical for the uptake of host cell cytosol (21). Critically, recent work has shown that an established marker of ART resistance, a kelch domain-containing protein called Kelch13 (K13) (44, 45) was essential in the early steps of the endocytic process (46).

A role for PPIs in the trafficking pathway of Hb from the host cell cytosol to the DV was previously hinted at by the observation that parasites incubated with the PI3P kinase (PI3K) inhibitor Wortmannin accumulated Hb-containing vesicles (47) and by the localization of PI3P on the DV membrane (17) and endosomal/Hb-filled vesicles (21). Importantly, none of the PI3P binding proteins characterized so far were shown to be implicated in this process. The Fab1, YOTB, Vac1, EEA1 (FYVE)-containing protein (PfFCP) localizes to the DV lumen and binds PI3P through its FYVE domain. However, this is not required for PfFCP localization to the DV so the role that this protein plays is unclear (48). While Autophagy-related protein 18 localizes to the DV membrane in a PI3P-dependent manner (49, 50), its conditional knockdown does not impact Hb trafficking, resulting instead in apicoplast inheritance defects (51). Other work has however suggested that it was also implicated in DV dynamics (49). Finally, PfHSP70-1 was recently shown to bind PI3P and to help stabilize the DV under heat stress (52).

With the goal of getting more insight into the role of PPIs in the asexual erythrocytic cycle of P. falciparum, we undertook knockout (KO) attempts on 24 genes coding for proteins putatively involved in PPI metabolism. These include lipid kinases, phosphatases and proteins with putative PPI-binding domains. Our results show that a majority of the candidates could not be inactivated suggesting that they are potentially essential for *in vitro* growth of the parasite. The analysis of a KO strain for a PX domain-containing *Plasmodium*-specific protein revealed a severe growth defect. Further characterization showed that the PX domain likely has the ability to specifically bind PI3P and that the protein localizes to the DV membrane and cytoplasmic vesicles that overlap with known markers of host cell-cytosol endocytosis. Using a variety of functional assays we provide several lines of evidence that the protein is potentially implicated in the trafficking pathway of Hb from the host cell to the DV.

## RESULTS

### Genetic inactivation attempts for putative phosphoinositide binding proteins and phosphoinositide enzymes.

In an attempt to delve into the roles of PPIs in the biology of the asexual stages of P. falciparum, we created a non-exhaustive list of potential PPI binding proteins by performing a Gene Text Search of the PlasmoDB database (www.plasmodb.org) with the followings terms: phosphoinositide, pleckstrin, FYVE, ENTH, PHOX, PX, and PDZ. This resulted in 13 proteins of which 2 contained FYVE domains, 1 Epsin N-terminal homology (ENTH) domain, 2 PX domains, 7 PH domains, and 1 PDZ domain (Table 1), in line with previous studies (16, 53). We also searched for putative PPI kinases and phosphatases using the search terms phosphoinositide, phosphatidylinositol, inositol-phosphate, PPIK, SAC, Syja_N, IP5K and identified six putative PPI kinases (54) and four putative PPI phosphatases. We also added the sole PPI synthase (55) to our list, for a total of 24 proteins. We next attempted to inactivate each of these genes using the selection-linked integration (SLI) for targeted gene disruption (TGD) method (56). Selection for integrants resulted in four scenarios. For seven genes, we could not recover parasites following neomycin (NEO) selection even with three independent attempts suggesting either that the gene cannot be inactivated or that the locus is not amenable to genetic modification. The transfectants that could be recovered were analyzed by PCR to detect the proper integration event and the absence of the wild-type allele (WT) (schematic Fig. S1A in the supplemental material). The PCR results revealed three different scenarios: i) correct integration of the targeting plasmid but WT allele still present (this suggests that there was a recombination event such as gene duplication so that a WT allele can still be expressed despite the original locus being disrupted; six genes, Appendix Fig. 1 at https://figshare.com/articles/figure/Appendix_Mukherjee_et_al_2021/16955911); ii) no integration and presence of the WT allele suggesting a recombination event outside of the target locus but nevertheless resulting in the expression of the NEO selection marker (6 genes, Appendix Fig. 2 at https://figshare.com/articles/figure/Appendix_Mukherjee_et_al_2021/16955911); iii) correct integration and absence of the WT allele, suggesting a successful knockout line (5 genes, Fig. S1B). Successful SLI leads to the expression of the N-terminus of the disrupted gene in fusion to green fluorescent protein (GFP) (56) and for all five KO lines, cytosolic fluorescence was indeed observed though it was sometimes very faint (Fig. S1C and Appendix Fig. 3 at https://figshare.com/articles/figure/Appendix_Mukherjee_et_al_2021/16955911). The inactivated genes coded for two PH domain-containing proteins, one PX domain-containing protein, a putative phosphatidylinositol-4-phosphate 5-kinase (PI(4,5)K) and a putative PPI phosphatase (Table 1). These results reveal that 21% of our putative PPI binding proteins and PPI enzymes are not essential for the asexual erythrocytic cycle.

**TABLE 1:** List of putative PPI-binding proteins and enzymes

PlasmodDB accession number	Domain	TGD^*a*^	Growth disadvantage	Pf Piggyback MIS/MFS (Zhang et al, 2018)	PbKO (Bushell et al, 2017)	TgKO (Sidik et al, 2016)
PF3D7_1459600	ENTH	NO	NA	0,12/−3	Yes	−4,16
PF3D7_1310300	FYVE	NO	NA	0,12/−3,11	No data	*Plasmodium* specific
PF3D7_1460100	FYVE (FCP)	NO	NA	0,12/−3,05	No data	−1,4
PF3D7_0307800	PDZ	NO	NA	0,15/−2,75	No data	−3,27
PF3D7_0414600	PH (APH)	NO	NA	0,68/−2,21	Slow growth	−5,53
PF3D7_1337700	PH (PfPH2)	NO	NA	0,13/−2,93	No data	0,26
PF3D7_1146100	PH	NO	NA	0,14/−2,89	No data	1,91
PF3D7_1131800	PH (OSBP)	Yes	Not significant	1/0	Slow growth	−6,07
PF3D7_1019600	PH	NO	NA	1/−0,2	Yes	Not in Tg
PF3D7_1361500	PH	Yes	Yes	0,93/−1,93	No data	1.55
PF3D7_1141300	PH	NO	NA	0,12/−3,05	No data	*Plasmodium* specific
PF3D7_0720700	PX (PfPX1)	Yes	Yes	1/−2,65	No data	*Plasmodium* specific
PF3D7_0704400	PX	NO	NA	0,26/−2,77	No data	−0,86
PF3D7_0311300	PI(3,4)K Class II	NO	NA	0.13/−2.97	Slow growth	−3,91
PF3D7_0515300	PI3K	NO	NA	0,24/−2,67	NO	−3,88
PF3D7_0110600	PI4,5K	NO	NA	0,2/−2,77	NO	−5,81
PF3D7_1129600	PI(4,5)K Set7/9 family)	Yes	No	1/−2,22	Yes	*Plasmodium* specific
PF3D7_0419900	PI4K Class III alpha	NO	NA	0,12/−3,01	Slow growth	*Plasmodium* specific
PF3D7_0509800	PI4K Class III beta	NO	NA	0,13/−3,01	NO	−3,6
PF3D7_1354200	Sac1 (PfSAC1)	NO	NA	1/−1,87	No data	−1,35
PF3D7_1111600	I5P_5	Yes	Yes	0,12/−2,89	No data	0,51
PF3D7_0705500	Sac1-I5P_5	NO	NA	0,14/−2,89	NO	*Plasmodium* specific
PF3D7_0802500	Sac1	NO	NA	0,95/−2,86	Yes	Not in Tg
PF3D7_1315600	PIP synthase	NO	NA	0,13/−3,21	No data	−3,57

*^a^*TGD, targeted gene disruption; NA, not applicable; MIS, mutagenesis index score; MFS, mutagenesis fitness score.

10.1128/mbio.03239-21.1 FIG S1 (A) Schematic for the SLI-TGD strategy. GOI: gene of interest. (B) PCR integration test results. Shown are agarose gel pictures of the PCR integration tests for the successful KO lines generated. 5′int: 5′ integration junction. 3′int: 3′ integration junction. WT: WT allele. The presence of amplicons in the 5′ and 3′int lanes and the absence of a WT allele confirm that the gene of interest has been successfully inactivated. The colored arrows show the primer pairs used for the PCR analysis. Results from one KO parasite line per gene is shown except for PF3D7_0720700 (PfPX1) where two lines are shown. bp: base pairs. (C, i) Anti-GFP Western blot on mixed stage PfPX1-SLI-TGD parasite extracts showing the expression of the PXdom-GFP fusion. (C, ii) Fluorescence microscopy showing that the GFP truncation resulting from SLI of the PfPX1 gene is cytosolic. Blue: Dapi-stained DNA. Download FIG S1, PDF file, 1.9 MB. Copyright © 2022 Mukherjee et al. 2022 Mukherjee et al. https://creativecommons.org/licenses/by/4.0/ This content is distributed under the terms of the Creative Commons Attribution 4.0 International license.

**FIG 1:**
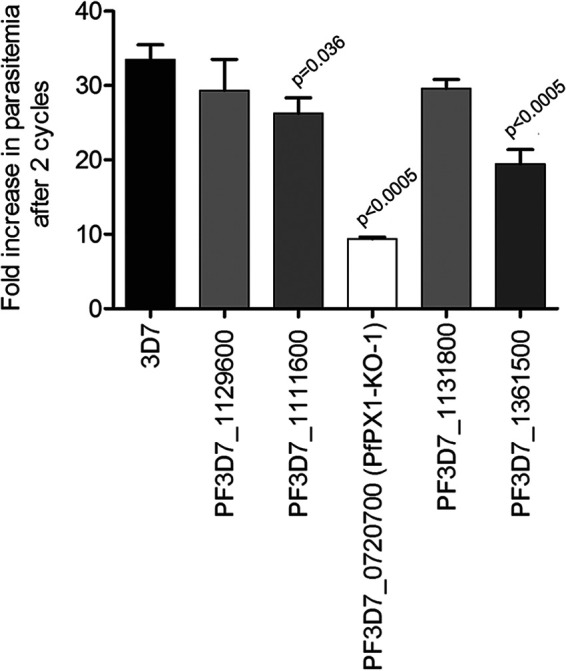
Analysis of the asexual growth of knockout lines of putative phosphoinositide binding proteins and phosphoinositide enzymes. Effect of inactivation of potential PPI-associated genes on asexual growth over two cycles by flow cytometry. Results are mean ± SEM of 3 to 6 biological replicates. Unpaired two-tailed *t* tests were performed to compare the parental strain (3D7) to all the KOs.

To determine if the fitness of the KO strains was affected, we evaluated their asexual growth over two replication cycles by flow cytometry. No difference was seen between the 3D7 WT control and the KOs of the putative PI(4,5)K PF3D7_1129600 and the PH and oxysterol binding domain protein PF3D7_1131800. However, growth defects were observed for the putative PPI phosphatase PF3D7_1111600, the PH domain protein PF3D7_1361500 and the PX domain protein PF3D7_0720700 (Fig. 1). Our data align well with the whole genome piggyback screen on P. falciparum apart for the PI(4,5)K where the fitness score of -2.22 suggested a growth defect (Table 1) (57). Because it showed the most severe growth defect of the 5 KO lines, we decided to further characterize the PX domain protein PF3D7_0720700 which will be referred to as PfPX1 from now on.

### Orthologues of PfPX1 are *Plasmodium* specific and contain a putative PI3P-binding sequence motif.

**FIG 2:**
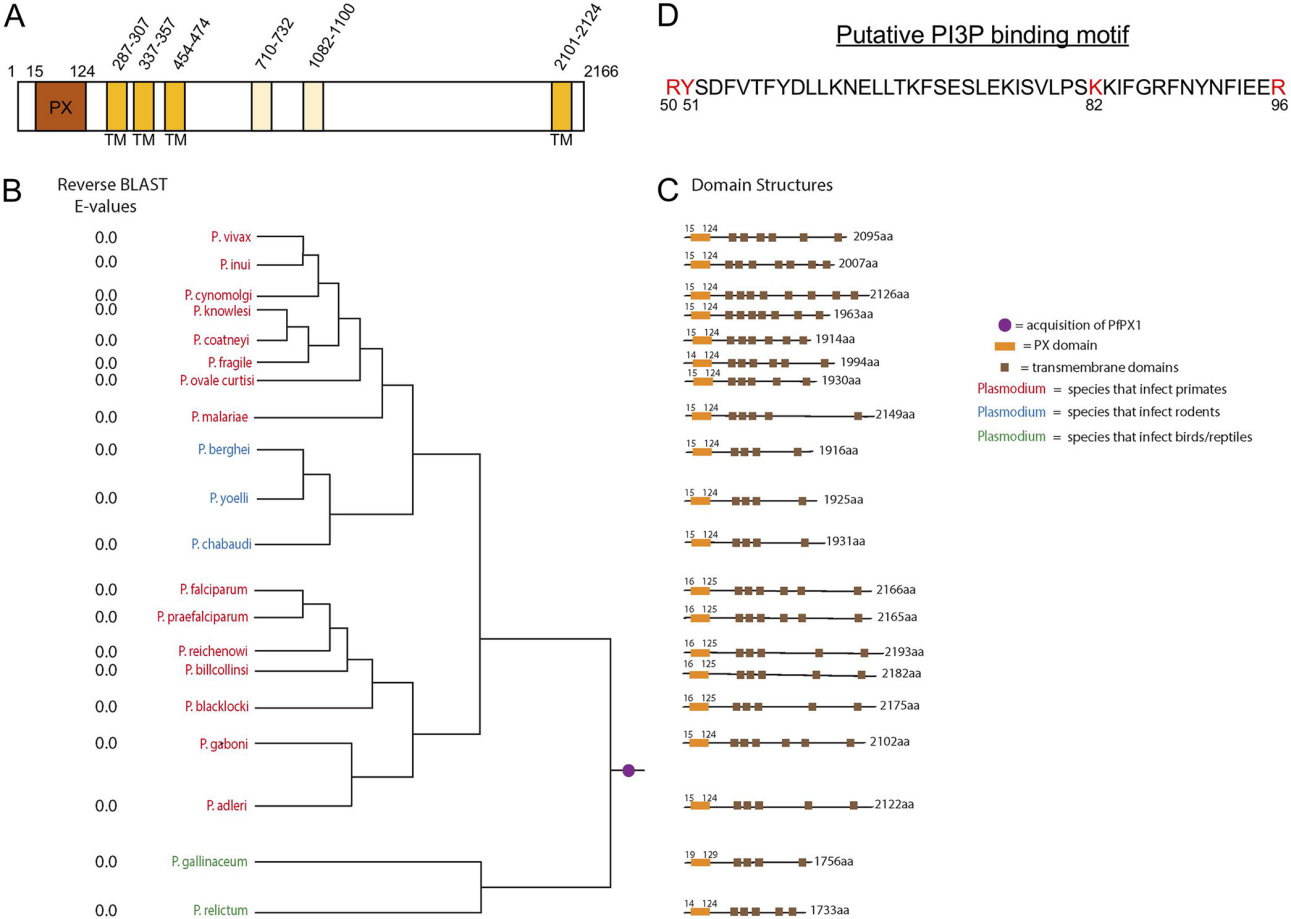
PfPX1 and the putative PI3P-binding PX domain are conserved in *Plasmodium* species. (A) Schematic of PfPX1 and putative domains. TM, transmembrane domain. The two lighter TMs have lower confidence scores than the other four. (B) PfPX1 proteins are conserved across all species of *Plasmodium*. The phylogenetic distribution demonstrates conservation of PfPX1 based on the E values for the candidate orthologue into the P. falciparum proteome (column reverse BLAST E values), regardless of infectious host (red, primates; blue, rodents; green, birds/reptiles). Relationships of taxa are based on (139). Purple circle represents the phylogenetic placement where the PfPX1 equivalent was acquired during evolution. (C) Schematic of PfPX1 displaying the position of the conserved PX domain and of putative transmembrane domains. Conserved domain structure is shown based on Phyre v2.0. aa, amino acids. Orange box: conserved PX domain. Brown boxes: putative transmembrane domains. (D) Putative PI3P binding motif in the PX domain of PfPX1. The four core binding residues are in red. The residues are numbered according to the full-length PfPX1 protein.

PfPX1 is a 2166 amino acid protein with a putative PX domain and 4 potential transmembrane (TM) domains based on PlasmoDB (www.plasmodb.org) (Fig. 2A). To assess the evolutionary distribution of the PfPX1 protein, we used both BLASTp and HMMer homology searching to look for PfPX1 orthologues in a wide variety of taxa, with emphasis on apicomplexan organisms and multiple *Plasmodium* species. The initial BLASTp searches using the full length PfPX1 as a query generated multiple positive results in organisms both within and outside the *Plasmodium* genus (data not shown). However, most of these protein hits outside of the *Plasmodium* genus also contained PX domains. In order to mitigate the possibility of false positives due to the conserved PX domain, we repeated the searches using PfPX1 but with the PX domain omitted. This retrieved only positive hits from proteomes of *Plasmodium* origin (Dataset 1, https://doi.org/10.6084/m9.figshare.14124542.v1). We therefore conclude that all *Plasmodium* species examined contained orthologues of PfPX1, regardless of infectious host (Fig. 2B), but that this is a genus-specific trait. Next, domain architecture was determined using Phyre v 2.0 and CDD on NCBI and shows conservation of the PX domain at the N-terminus of the protein in all orthologues examined (Fig. 2C). PfPX1 is predicted to have 4 putative TMs in PlasmoDB. To determine whether these were conserved in the orthologues, we used PHYRE and TMpred and found that all orthologues possessed between four and eight putative TMs (Fig. 2C). Interestingly, six TMs were detected in PfPX1 compared with the four in PlasmoDB however the additional TMs (TMs 4 and 5) had much lower confidence scores than the other four (Dataset 2, https://doi.org/10.6084/m9.figshare.16837750.v1). Whether there are actually four or 6 TMs, this wouldn’t impact the protein’s topology (see below).

PX domains have a core fold consisting of three antiparallel ß-strands followed by three α-helices (58, 59). Recent work has revealed that PX domains with the ability to bind PI3P possess a core of four critical residues: an arginine followed by a tyrosine at the junction between ß-strand 3 and α-helix 1, a lysine in a motif called the PPK loop (ΨPxxPxK, where Ψ is a large aliphatic amino acid like valine, isoleucine, leucine or methionine) and an arginine within the second α-helix (60). Analysis of the sequence of the PX domain of PfPX1 revealed the presence of all four core PI3P binding residues though the first P of the PPK loop is replaced by a serine (ISVLPK, Fig. 2D). We next wanted to determine if the four core PI3P-binding residues identified within the PX domain of PfPX1 were conserved between all orthologues. As seen in Figure S2 in the supplemental material, the three critical positively charged residues (R50, K82, and R96) are conserved in all orthologues.

10.1128/mbio.03239-21.2 FIG S2 Alignment showing the conservation of the core PI3P binding residues in the PfPX1 orthologues of all *Plasmodium* species surveyed. PI3P binding motif R_50_Y_51_K_82_R_96_ was aligned with PfPX1 orthologues from *Plasmodium* spp. to evaluate binding motif conservation. The R_50_Y_51_K_82_R_96_ motif is conserved within the members of Laverania, while all other species contain a similar R_50_F_51_K_82_R_96_motif. The location of the PX domain structures were determined using Phyre v 2.0 and CDD on NCBI. Arrows: signifies positioning of the R_50_Y_51_K_82_R_96_/R_50_F_51_K_82_R_96_motifs. Download FIG S2, PDF file, 1.8 MB. Copyright © 2022 Mukherjee et al. 2022 Mukherjee et al. https://creativecommons.org/licenses/by/4.0/ This content is distributed under the terms of the Creative Commons Attribution 4.0 International license.

### The PX domain of PfPX1 likely binds to PI3P.

**FIG 3:**
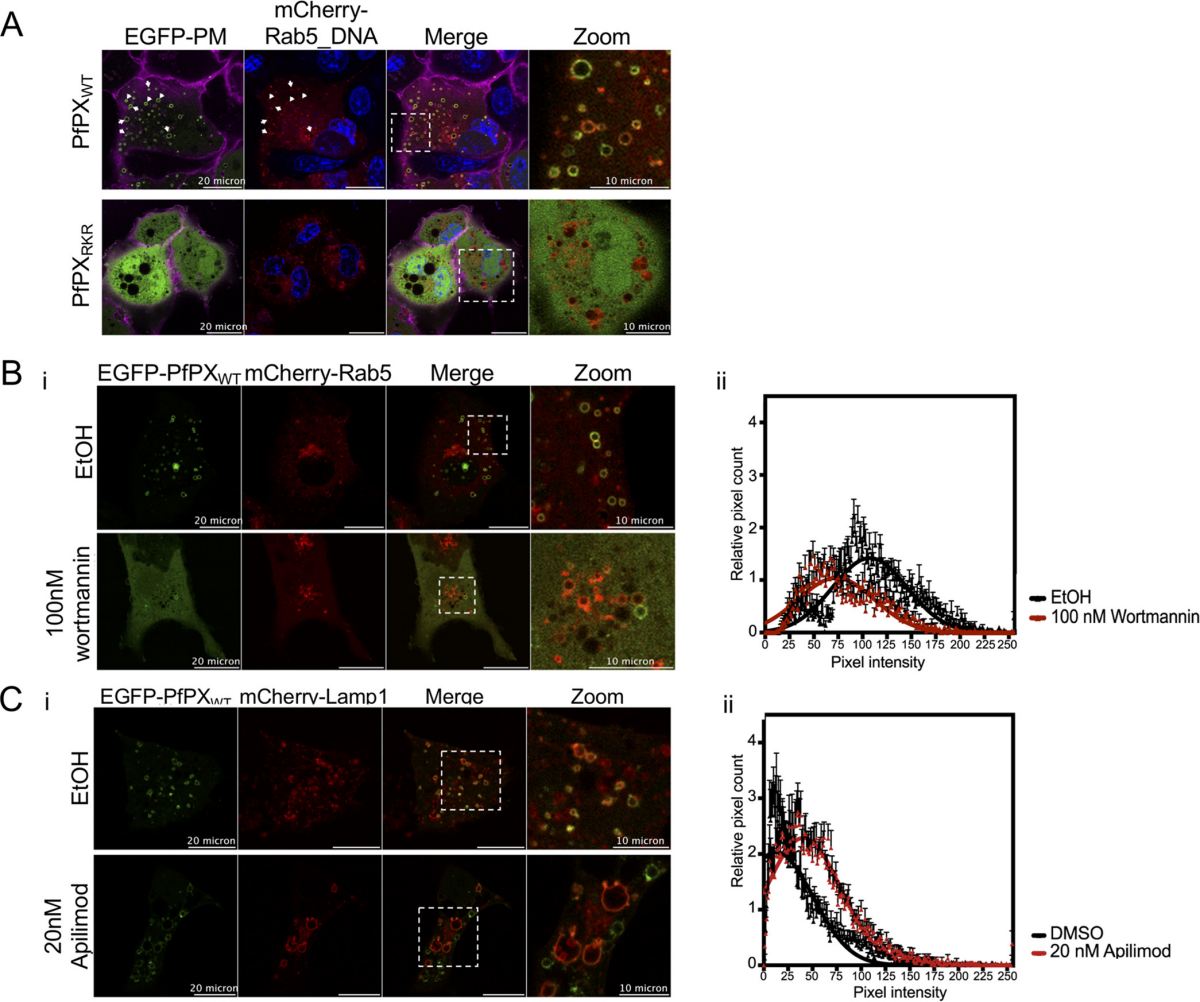
The PX domain of PfPX1 likely binds selectively to PI3P. (A) Representative images of Cos-7 cells co-transfected with EGFP-PfPX_WT_ or EGFP-PfPX_RKR_ and mCherry-tagged early endosome marker Rab5. Blue: Hoechst-stained DNA. Magenta: plasma membrane stained with wheat-germ hemagglutinin-Alexa fluor 647. White arrows show colocalization of EGFP signal with mCherry signal indicating PfPX_WT_ binding to early endosomes. Zoom panel corresponds to the insets showed in Merge panel. (B) Representative confocal images of Cos-7 transfected with EGFP-PfPX_WT_ treated with ethanol (EtOH) or 100 nM Wortmannin. EGFP fluorescence is more cytosolic and reduced at mCherry-Rab5 positive structures in Wortmannin-treated cells (i). This reduction of EGFP signal is represented as histogram of intensity values (ii) indicating the reduction of pixel counts and intensity at mCherry-Rab5 positive structures upon Wortmannin treatment. (C) Representative confocal images of Cos-7 transfected with EGFP-PfPX_WT_ treated with DMSO or 20 nM Apilimod (i). The EGFP-signal is represented as histogram of intensity values (ii) indicating a slight increase of pixel intensity at mCherry-LAMP-1 positive structures upon PIKfyve inhibition. The data in panels B and C are shown as the mean ± standard deviation with Gaussian fitting from three independent replicates, with a total of 40 to 60 vesicles.

To determine the PPI selectivity of the PX domain of PfPX1, we attempted to recombinantly express the N-terminal 151 amino acids of PfPX1 containing the entire predicted PX domain and a mutant version with residues R50, K82 and R96 mutated to glutamine. However, we did not succeed in obtaining stable soluble proteins. To overcome this issue, we generated mammalian expression vectors encoding fluorescently labeled PfPX domains (EGFP-PfPX_WT_ and EGFP-PfPX_RKR_) to express them in mammalian cells, a validated alternative methodology used to determine PPI binding specificities (61–63). Mammalian intracellular endocytic membranes have unique and dynamic PPI compositions and the presence of PI3P on the cytoplasmic face of early endosomes is well-established (64–66). To verify the ability of the PfPX domain to selectively bind PI3P-containing membranes, we assessed the co-localization of EGFP-PfPX_WT_ domain with mCherry labeled endocytic pathway markers in the mammalian cell line Cos-7 (Rab5: Ras-related protein marker of early endosome, Rab9a: late endosome, Rab11: recycling endosome, LAMP-1: Lysosomal-associated membrane protein 1). EGFP-PX_WT_ co-localized with membranes positive for Rab5 while it showed a poor co-localization with Rab9a and Lamp1-positive membranes and no co-localization with Rab11-membranes (Fig. 3A and Fig. S3 in the supplemental material). Given that Rab5 is a well-established marker of early endosomes enriched in PI3P, this demonstrates the specific enrichment of EGFP-PX_WT_ to PI3P-containing membranes (64). The analysis of EGFP-PX_RKR_ mutant confirmed that the mutated residues are required for PI3P-dependent localization since it was unable to localize to any intracellular membranes (Fig. 3A). Next, we chemically reduced the level of PI3P on intracellular membranes using Wortmannin, a potent and selective PI3-kinase inhibitor. In the presence of Wortmannin, EGFP-PX_WT_ showed a reduced binding to intracellular membranes as measured by a decrease of 50% of pixel counts and intensity on Rab5-positive membranes (Fig. 3B). As a positive control for PI3P-dependent localization, we used a fusion of EGFP with the PX domain from p40^phox^ and this showed similar results where EGFP-p40*^phox^* was found mostly cytoplasmic in the presence of Wortmannin (Fig. S4A). Lastly, to further confirm that EGFP-PX_WT_ enrichment at early endosomal membrane was indeed PI3P-dependent, we treated Cos-7 cells with 20 nM Apilimod, a very potent and selective PIKfyve (PI3P5-kinase) inhibitor (67, 68) that reduces PI(3,5)P_2_ and increases PI3P levels with disruption of endo-lysosomal integrity (61). First, we observed an increase of LAMP-1-vesicle volume suggesting an endolysosome vacuolation effect in treated cells as expected (Fig. S4B). However, EGFP-PX_WT_ localization was unaffected by Apilimod treatment and the fluorescent protein was retained on LAMP-1-associated membranes along with an increase of pixel intensity, five times more than DMSO treatment (Fig. 3C). Collectively, our data confirm that PfPX domain association to endosomal and endolysosomal membranes is dependent on PI3-kinase activity and likely its selective interaction with PI3P in mammalian cells.

10.1128/mbio.03239-21.3 FIG S3 Representative confocal images of Cos-7 cells co-transfected with EGFP-PfPX_WT_ and different mCherry-tagged endocytic markers Rab9a (late endosome), Rab11 (recycling endosome), LAMP-1 (lysosome). Blue: Hoechst-stained DNA. Magenta: plasma membrane stained with wheat-germ hemagglutinin-Alexa fluor 647. PM: Plasma membrane. Zoom panel corresponds to the insets showed in Merge panel. Download FIG S3, PDF file, 2.3 MB. Copyright © 2022 Mukherjee et al. 2022 Mukherjee et al. https://creativecommons.org/licenses/by/4.0/ This content is distributed under the terms of the Creative Commons Attribution 4.0 International license.

10.1128/mbio.03239-21.4 FIG S4 (A) Representative confocal images of Cos-7 cells transfected with EGFP-p40^phox^ indicating bright fluorescence that accumulated with Rab5-mCherry fluorescence as described previously (140) in control ethanol (EtOH) but in not 100 nM Wortmannin treated cells where EGFP-fluorescence is more cytosolic and reduced at mCherry-Rab5 positive structures (i). This reduction of EGFP-signal is represented as histogram of intensity values (ii) indicating the reduction of pixel counts and intensity at mCherry-Rab5 positive structures upon Wortmannin treatment. The data shown are the mean ± standard deviation with Gaussian fitting from three independent replicates, with a total of 37-40 vesicles. (B) Quantification of individual endo/lysosome volume in Cos-7 cells transfected with mCherry-LAMP-1 treated with DMSO (control) or 20 nM Apilimod. The data are shown are the mean ± standard deviation from three independent replicates, with a total of 250 endo/lysosomes. ****, P < 0.0001 compared with control condition using unpaired Student’s *t* test. Download FIG S4, PDF file, 0.2 MB. Copyright © 2022 Mukherjee et al. 2022 Mukherjee et al. https://creativecommons.org/licenses/by/4.0/ This content is distributed under the terms of the Creative Commons Attribution 4.0 International license.

As stated above, PI3P was previously shown to label the P. falciparum DV membrane (17, 20, 69). Our results showing that the PX domain of PfPX1 selectively bound to PI3P in mammalian cells thus led us to hypothesize that expressing the domain in P. falciparum would lead to the labeling of the DV membrane. Indeed, transfection of a plasmid expressing the isolated PX domain fused to GFP resulted in a nice labeling around the DV in addition to potential vesicular structures (Fig. S5A in the supplemental material). Furthermore, treatment of the parasites with the specific PI3K inhibitor LY294002 resulted in a 4-fold decrease in the number of DV-labeled cells which was not observed with LY303511, a derivative of LY294002 inactive against PI3K (70), or the DMSO control (Fig. S5B) again suggesting that PfPX1 likely selectively binds to PI3P. Taken together, our results obtained from both mammalian cells and P. falciparum strongly suggest that the PX domain of PfPX1 likely selectively binds PI3P.

10.1128/mbio.03239-21.5 FIG S5 The PX domain of PfPX1 localizes to the digestive vacuole of P. falciparum in a PI3P-dependent manner. (A, i) Western blot on mixed-stage parasites showing the expression of the PX domain fused to GFP. (A, ii) Live cell imaging of a parasite line expressing the PX domain fused to GFP (PXdom-GFP) showing the fluorescence patterns observed. Dapi: parasite nuclei. (B) Quantification of the parasites with DV-associated fluorescence or cytosolically diffused fluorescence following treatment with either 250 μM of the PI3K inhibitor LY294002 (IC90), 50 μM of its inactive equivalent LY303511 (IC90) or 0.25% DMSO. Graph shows treatment with 250 μM LY294002 decreases the DV-associated signal. (LY294002, n = 24; LY303511, n = 28; 0.25% DMSO, n = 19). Results from one representative experiment shown. Download FIG S5, PDF file, 0.8 MB. Copyright © 2022 Mukherjee et al. 2022 Mukherjee et al. https://creativecommons.org/licenses/by/4.0/ This content is distributed under the terms of the Creative Commons Attribution 4.0 International license.

### PfPX1 is constitutively expressed and localizes to punctate structures and the digestive vacuole membrane.

**FIG 4:**
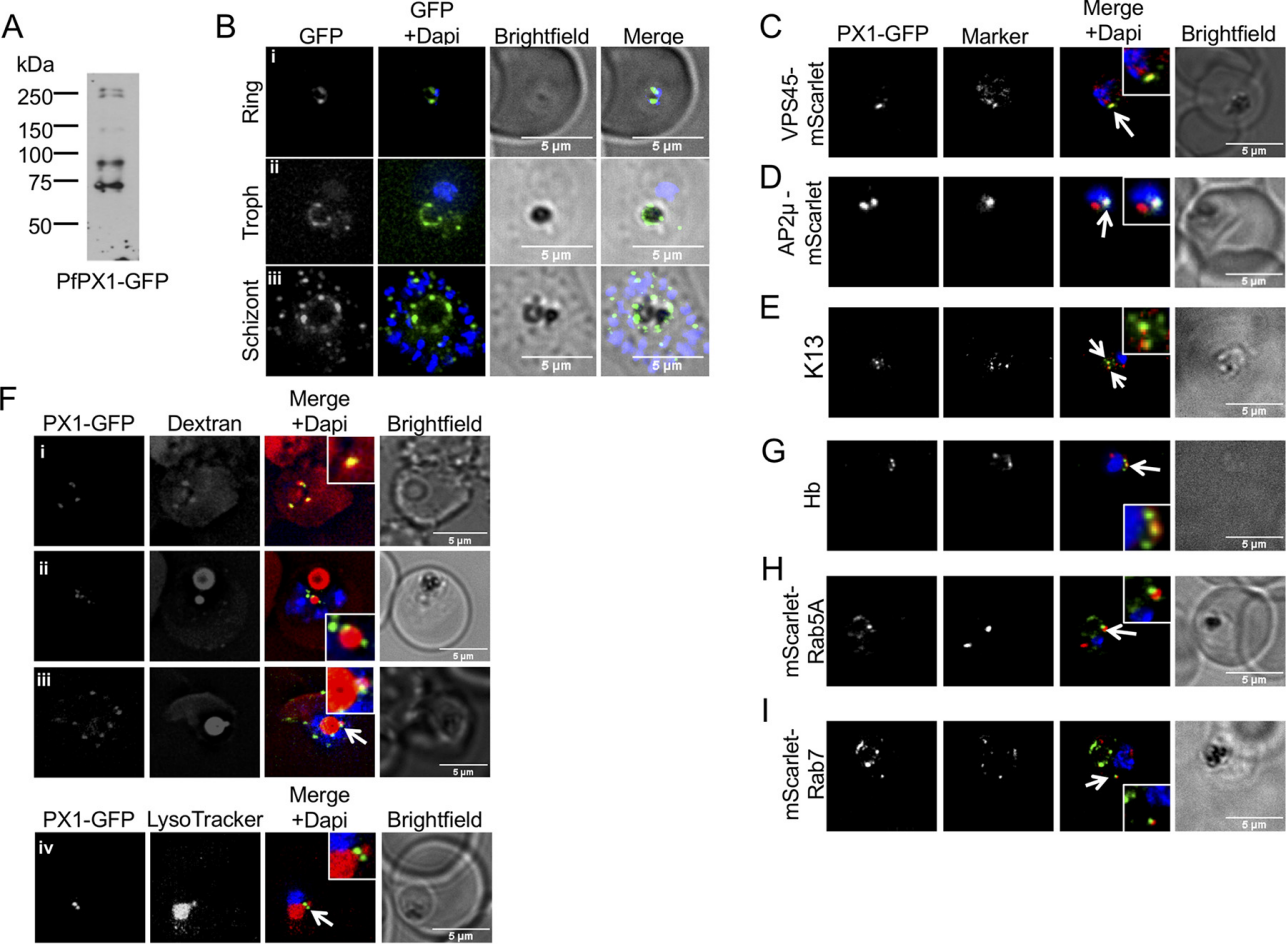
Subcellular localization of PfPX1-GFP. (A) Anti-GFP western blot of mixed stage PfPX1-GFP parasites showing the expression of the fusion protein. (B) Live cell imaging of the PfPX1-GFP parasite line at 3 different stages. Troph: trophozoite. (C) Colocalization of PfPX1-GFP with the endolysosomal marker PfVPS45-mScarlet. White arrow shows the overlapping foci. (D) Colocalization of PfPX1-GFP with PfAP2μ-mScarlet. White arrow shows the overlapping foci. (E) Colocalization of PfPX1-GFP with PfKelch13 (K13) using an anti-PfK13 antibody. White arrow shows the overlapping foci. (F) PfPX1-GFP in red blood cells preloaded with dextran Alexa-594. (F, iv) Colocalization of PfPX1-GFP with LysoTracker. White arrow in iii and iv show the DV extension. Single optical slices shown. (G) Colocalization of PfPX1-GFP with hemoglobin-filled structures. White arrow shows the overlapping foci. (H) Colocalization of PfPX1-GFP with mScarlet-PfRab5a. White arrow shows the juxtaposed foci. (I) Colocalization of PfPX1-GFP with mScarlet-PfRab7. White arrow shows the juxtaposed foci. Dapi: parasite nuclei.

To determine the subcellular localization of PfPX1, we endogenously tagged its gene with GFP or with spaghetti monster HA (smHA) using SLI (Fig. S6 in the supplemental material) and monitored the localization throughout the asexual cycle of P. falciparum using one clonal line for each. Western blots on mixed (PfPX1-GFP) or schizont stage (PfPX1-smHA) parasite extracts detected multiple bands with the highest ones being above 250 kDa, in line with the expected size of the full-length proteins of around 285 kDa for PfPX1-GFP and 297 kDa for PfPX1-smHA (Fig. 4A and Fig. S7C in the supplemental material), suggesting that PfPX1 is proteolytically processed. In ring stages, one to a few foci of fluorescence were seen but as the parasite matured, numerous foci appeared with a substantial proportion surrounding the DV (Fig. 4B and Fig. S7A). This was especially striking in trophozoites and schizonts of the PfPX1-GFP line where in addition to the foci, the fluorescence could be seen distributed around the DV (Fig. 4Bii and iii), perhaps because these were live cells that had not gone through fixation/permeabilization.

10.1128/mbio.03239-21.6 FIG S6 (A) Schematic of the strategy used to generate the PfPX1-GFP and PfPX1-smHA parasites lines by SLI. (B) PCR tests confirming the integration of the pSLI-PfPX1-GFP plasmid at the c-terminus of the PfPX1 gene and the absence of a WT allele. (C) PCR tests confirming the integration of the pSLI-PfPX1-smHA plasmid at the c-terminus of the PfPX1 gene and the absence of a WT allele. Colored arrows show primer pairs used for PCR. 5’int: 5’ integration junction. 3’int: 3’ integration junction. WT: WT allele. Download FIG S6, PDF file, 2.0 MB. Copyright © 2022 Mukherjee et al. 2022 Mukherjee et al. https://creativecommons.org/licenses/by/4.0/ This content is distributed under the terms of the Creative Commons Attribution 4.0 International license.

10.1128/mbio.03239-21.7 FIG S7 Subcellular localization of PfPX1-smHA/GFP. (A) Immunofluorescence detection of PfPX1-smHA in blood stage parasites. h: hours post invasion. Dapi: parasite nuclei. Yellow arrows: Foci at/close to the digestive vacuole membrane. (B) Colocalization of PfPX1-smHA with the ER marker PfBiP and the Golgi apparatus marker PfERD2. Dapi: parasite nuclei. (C, i) Western blot showing expression of PfPX1-smHA at different time points of the asexual cycle. h: hours post invasion. Red (full length) and yellow (processed) rectangles show areas used for quantification by densitometry analyses in Cii. (C, ii) Quantification of the proportion of the full length and processed PfPX1-smHA at each time point. (D) Western blot showing the amount of full length (red rectangles) and processed PfPX1-smHA (yellow rectangles) in the vesicular fraction and the digestive vacuole-containing fraction. An antibody against PfPlasmepsin 2 (PfPM2) was used as a control for increased processing in the digestive vacuole. Download FIG S7, PDF file, 1.1 MB. Copyright © 2022 Mukherjee et al. 2022 Mukherjee et al. https://creativecommons.org/licenses/by/4.0/ This content is distributed under the terms of the Creative Commons Attribution 4.0 International license.

To better characterize the localization of PfPX1, colocalization assays with a variety of intracellular structure markers were performed. In a few instances, some overlap was sometimes seen between a focus of PfPX1 fluorescence and the endoplasmic reticulum marker BiP (Fig. S7B in the supplemental material). Although some of the PfPX1 foci were sometimes close to the Golgi apparatus marker ERD2, no overlap was observed (Fig. S7B). The punctate pattern of PfPX1 and its localization around the DV led us to hypothesize that the foci might correspond to host cell cytosol (HCC)-filled vesicles on their way to be delivered to the DV. We thus tested whether PfPX1 colocalized with PfVPS45, PfAP2μ and PfK13, proteins recently shown to be involved in HCC endocytosis (21, 46, 71–73). Despite low levels of fluorescence in the parasite lines overexpressing PfVPS45 and PfAP2 fused to mScarlet, parasites where some PfPX1-GFP foci overlapped with each protein could be observed (Fig. 4C and D). In addition, colocalization could also be seen between some PfPX1-GFP foci and PfK13 by IFA with an anti-PfK13 antibody (Fig. 4E). These results suggest that PfPX1 might potentially play a role in the HCC trafficking pathway. It is interesting that for all markers tested, only a few of the PfPX1-GFP and marker foci overlapped. This had previously been described in a study looking at the colocalization between PfPlasmepsin II-GFP and TMR-dextran-filled vesicles (40). To directly determine whether the PfPX1 foci corresponded to vesicles filled with HCC, we preloaded erythrocytes with Alexa-Fluor-594 labeled dextran and infected them with the PfPX1-GFP parasite line. In ring stage parasites, the PfPX1 foci overlapped with dextran foci that looked to be at or close to the plasma membrane (Fig. 4Fi). These are potentially acidic peripheral compartments likely derived from cytostomal invaginations and previously proposed to coalesce and form the single acidic DV^40^. Unfortunately, we could never detect dextran vesicles in later stage parasites which could be due to the very strong labeling of the DV. However, additional dextran filled structures were often seen with some resembling extensions of the DV whilst others looked to be separate from the organelle. These might perhaps represent lobes of the recently described multilobed DV^49^. Of interest, anytime these structures were seen, PfPX1-GFP foci were always associated/in close proximity (Fig. 4Fii and iii). This was also seen when parasites were labeled with the lysosome marker dye LysoTracker, known to accumulate inside acidic organelles like the DV (74) (Fig. 4Fiv). Next, we performed IFAs with an anti-Hb antibody and these showed that some PfPX1-GFP foci colocalized with Hb, consistent with the results we obtained with dextran-preloaded RBCs (Fig. 4G). Finally, we tested the potential colocalization of PfPX1-GFP with the endolysosomal markers Rab5a and Rab7, proteins potentially implicated in HCC trafficking and DV dynamics (75, 76). Whilst neither of the markers overlapped with PfPX1-GFP, there were often some juxtaposed foci (Fig. 4G and I). Taken together, the localization data suggest that PfPX1 could be potentially implicated in the trafficking of HCC to the DV.

Of note, the localization pattern we observed with the isolated PX domain of PfPX1 is similar to the full-length protein (Fig. S5Aii in the supplemental material versus Fig. 4Bii) suggesting that the PX domain contains all the information needed for proper targeting. However, the truncated protein containing the first 103 amino acids of PfPX1 and lacking the last 21 residues of the PX domain (resulting from the generation of the PfPX1-KO line by SLI) is mislocalized to the cytosol (Fig. S1Cii).

To further evaluate the expression profile of PfPX1 throughout the asexual cycle, we next performed a time course of expression by Western blot using protein extracts of the PfPX1-smHA line taken throughout the cycle. In line with the microscopy results, we detected PfPX1-smHA at all analyzed time points (Fig. S7Ci in the supplemental material). As stated above, in addition to the major band above 250kDa, several smaller bands were seen, however their intensity relative to the full-length band seemed to increase as the parasites matured. This was confirmed by quantifying the signals by densitometry (Fig. S7Cii). It is important to note that since the smHA tag is at the C-terminus of the protein, all the bands we detect by Western blot with the anti-HA antibody contain the C-terminus. Because of the increased number of foci seemingly associated with the DV, we hypothesized that perhaps the degradation of PfPX1 was a result of the activity of DV proteases as was previously observed with PfPlasmepsin-2 (PM2) tagged with GFP (77, 78). To look into this, we lysed PfPX1-smHA parasites by nitrogen cavitation and separated the vesicular fraction from the organellar fraction containing DVs. Western blotting revealed that in the vesicular fraction, a higher amount of full-length PfPX1-smHA is present compared to the degradation products and this is the same for PfPM2 (Fig. S7D). Globally, our data thus suggest that PfPX1 is found as a full-length protein in vesicles and is degraded once these vesicles fuze with the DV membrane.

PfPX1 possesses 6 putative TMs and topology prediction using TMpred suggested that the N-terminus containing the PX domain and the C-terminal tail face the cytosol whilst the large disordered region would be intra-luminal (Fig. S8A in the supplemental material). To investigate this experimentally, proteinase K protection assays were performed on schizont stage PfPX1-smHA parasites. Following saponin treatment to release the erythrocyte cytosol, the parasites were treated with digitonin to selectively permeabilize the plasma membrane whilst leaving intracellular organelles intact. Subsequent treatment with proteinase K results in the degradation of proteins facing the cytosol whilst the lumenal proteins are preserved. The disappearance of the PfPX1-smHA Western blot signal in parasite extracts treated with proteinase K revealed that the smHA tagged C-terminus of PfPX1 was facing the cytosol. As expected, the luminal ER protein PfBiP was protected from degradation (Fig. S8B). Based on the *in silico* topology analysis, the N-terminus containing the PX domain should also face the cytosol, though in absence of an N-terminal tag or a specific antibody, we cannot experimentally determine if this is the case. However, this is likely if the PX domain is to be able to interact with PI3P on intracytosolic membranes.

10.1128/mbio.03239-21.8 FIG S8 Topology analysis of PfPX1-smHA. (A) *In silico* prediction of the topology of PfPX1-smHA. TM: transmembrane domain. Cartoon not to scale. (B) Proteinase K protection assay. PfPX1-smHA schizonts were treated with either saponin, saponin-digitonin or saponin-digitonin-proteinase K and probed with an anti-HA antibody. PfBiP was use as a control for an intra-lumenal protein. Download FIG S8, PDF file, 0.2 MB. Copyright © 2022 Mukherjee et al. 2022 Mukherjee et al. https://creativecommons.org/licenses/by/4.0/ This content is distributed under the terms of the Creative Commons Attribution 4.0 International license.

### Inactivation of PfPX1 leads to a defect at the ring/trophozoite stages in a portion of the parasite population.

**FIG 5:**
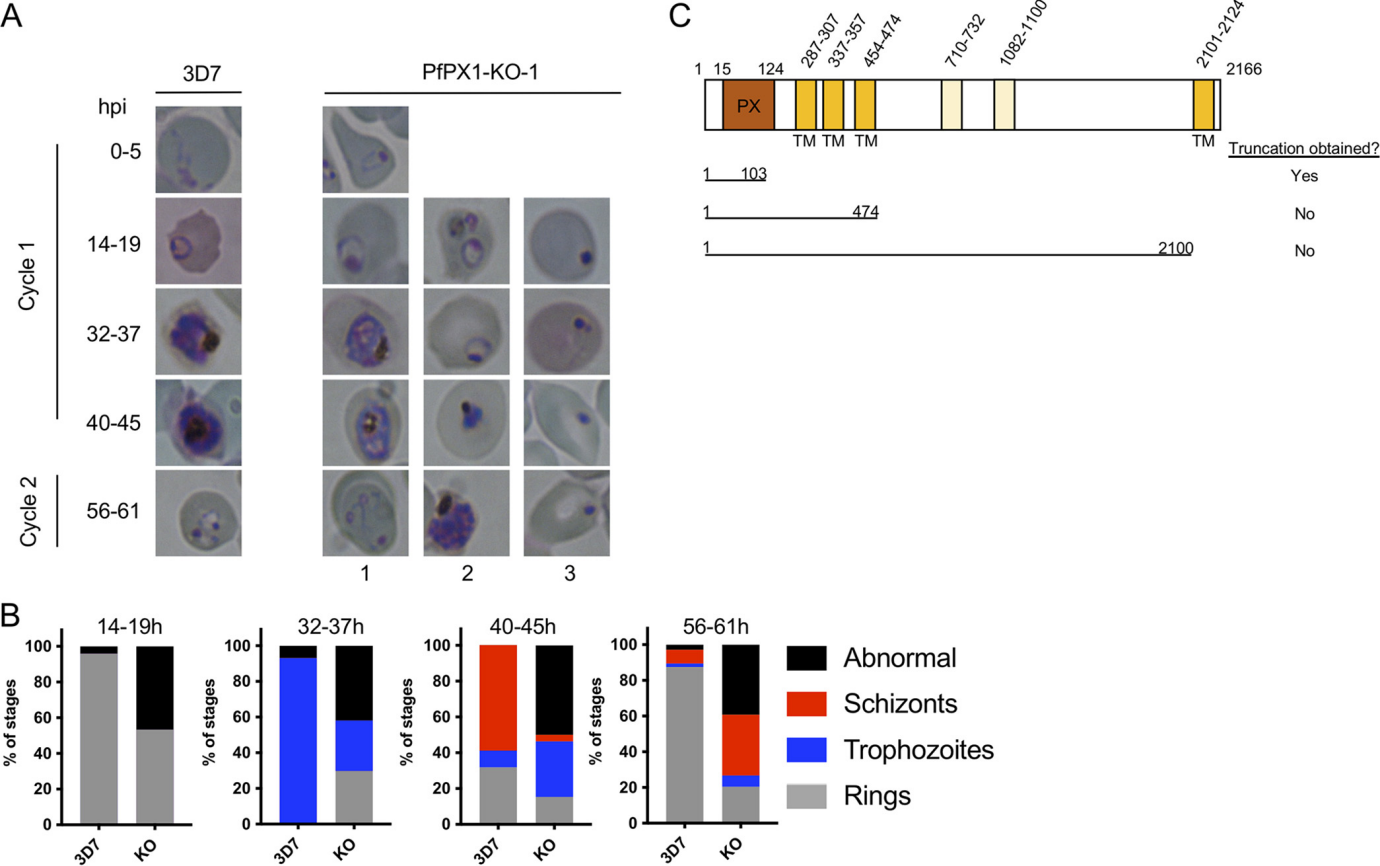
Phenotypic analysis of the growth of the PfPX1 knockout line. (A) Light microscopy of Giemsa-stained smears of synchronous 3D7 WT and PfPX1-KO-1 lines taken at different times throughout the blood stage over one and a half cycles. hpi: hours post invasion. Column 1 shows parasites with normal development. Column 2: Delayed parasites. Column 3: Abnormal parasites. (B) Quantification of the parasite stages in development across a cycle and a half. 300 parasites were scored for rings, trophozoites, schizonts and abnormal at each time point for each line and represented as % of stages. Results from one representative experiment of three independent experiments are shown. (C) Schematic representing the attempted PfPX1 truncations. TM: transmembrane domain. Cartoon not to scale.

To try to decipher the function of PfPX1 and further evaluate where in the asexual cycle the growth defect stemming from its inactivation occurred, we assessed the growth of 2 clones of tightly synchronized PfPX1-KO parasites (KOs) by Giemsa-stained smears. At the late ring stage, more than 50% of the KO parasites were pyknotics whilst another looked like healthy rings (Fig. 5A and B: 3D7, 96% healthy/4% pyknotic versus KO: 43% healthy/57% pyknotic). Subsequently, a large fraction of the KOs did not develop at the same rate as the parental 3D7 line. When the 3D7 were trophozoites, at 32-37h, around half of the population of KOs (not counting pyknotics) was delayed and were still rings (Fig. 5B, 32–37 h: 3D7, 0% rings/100% trophozoites versus KO around 50% rings/50% trophozoites). Similarly, when the 3D7 control parasites were mature schizonts and early rings, some KOs had only progressed to the trophozoite stage (Fig. 5B, 40–45 h: 3D7, 9% trophozoites/59% schizonts/32% rings versus KO 62% trophozoites/8% schizonts/30% rings). Finally, at 56–61 h when 3D7 were mostly mid rings in the next cycle, 68% of KOs were still schizonts. Taken together, these results suggest that the absence of PfPX1 does not have the same effect in every single cell, ranging from seemingly no effect at all, to a slowing of growth, to the death of the parasite at the ring stage. Importantly, we noticed that after several weeks in culture, the growth defect of the PfPX1-KO parasites did not seem qualitatively as severe which suggests that the parasites were potentially in the process of adapting. Because of this, all subsequent assays were performed with PfPX1-KO lines that had spent a maximum of 4 weeks in culture after the initial thawing.

To try to gain further insight into the function of PfPX1, we attempted to generate transgenic parasites expressing additional truncations of the protein: one with the N-terminus containing the PX domain up to right after the third putative transmembrane domain (amino acids 1 to 474), before a disordered region, and another where only the final transmembrane domain and C-terminal region would be deleted (amino acids 1 to 2100) (Fig. 5C). Unfortunately, we could never recover transgenics with either of the modifications. Our ability to generate the PfPX1-KO line, where only the first 103 amino acids of the protein are expressed, resulting in the truncation of the last 27 residues of the PX domain and the mislocalization of the protein to the cytosol (Fig. S1C), suggests that the parasite might not tolerate a properly localized nonfunctional PfPX1.

### Inactivation of PfPX1 results in increased accumulation of cytosolic Hb but has no effect on Hb digestion and hemozoin formation in the digestive vacuole.

**FIG 6:**
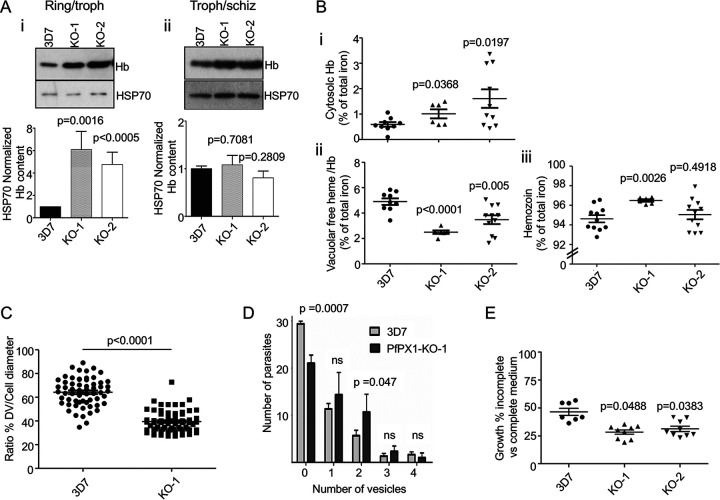
PfPX1 knockout lines accumulate more Hb and have a pronounced growth defect in amino acid-restricted medium. (A) Western blot analysis of Hb from saponin-freed parasites harvested at the trophozoite (3D7) and ring/trophozoite (PfPX1-KOs) stages (i) and schizont (3D7) and trophozoites/schizont (PfPX1-KOs) stages (ii). HSP70 was used as a loading control for normalization. Bar graphs represent the levels of Hb normalized to the HSP70 levels as determined by densitometry analysis. One-way ANOVA was performed for statistical evaluation. Results are expressed as mean ± SEM from 3 independent biological replicates. Troph: trophozoites. Schiz: schizonts. (B) Heme fractionation assay comparing the 3D7 WT to two PfPX1 KO lines. (B, i) Proportion of total cellular iron made up of cytosolic hemoglobin (3D7, *n* = 9; PfPX1-KO-1, *n* = 6; PfPX1-KO-2, *n* = 9). (B, ii) Proportion of total cellular iron made up of digestive vacuole-associated hemoglobin and free heme (3D7, *n* = 9; PfPX1-KO-1, *n* = 6; PfPX1-KO-2, *n* = 9). (B, iii) Proportion of total cellular iron made up of hemozoin (3D7, *n* = 9; PfPX1-KO-1, *n* = 6; PfPX1-KO-2, *n* = 9). Statistics were done using two-tailed unpaired *t* tests. (C) Digestive vacuole bloating assay. Ratio of the digestive vacuole diameter versus parasite diameter after an 8-h incubation with E64 for 3D7 and PfPX1-KO-1 lines. Each data point represents one cell. Values from three independent biological replicates are pooled together. A minimum of 20 cells per replicates were measured. A two-tailed unpaired *t* test was performed for statistical analysis. (D) Quantification of the number of Hb-containing vesicles as determined by IFA using an anti-Hb antibody on trophozoite (3D7) and ring/trophozoite (PfPX1-KO-1) stages. Results are expressed as mean ± SEM from a total of 50 parasites each from three independent biological replicates. Two-tailed unpaired *t* tests were performed for statistical evaluation. ns: not significant. (E) Evaluation of parasite growth in an amino-acid deficient medium. Tightly synchronous ring stage parasites were grown for two cycles in either complete or amino-acid deficient (containing only cysteine, glutamate, glutamine, methionine and isoleucine) medium. Data show the % of growth in amino acid-deficient medium compared to complete medium. Mean ± SEM from a minimum of seven independent experiments is shown. Unpaired *t* tests were performed for statistical evaluations.

**FIG 7:**
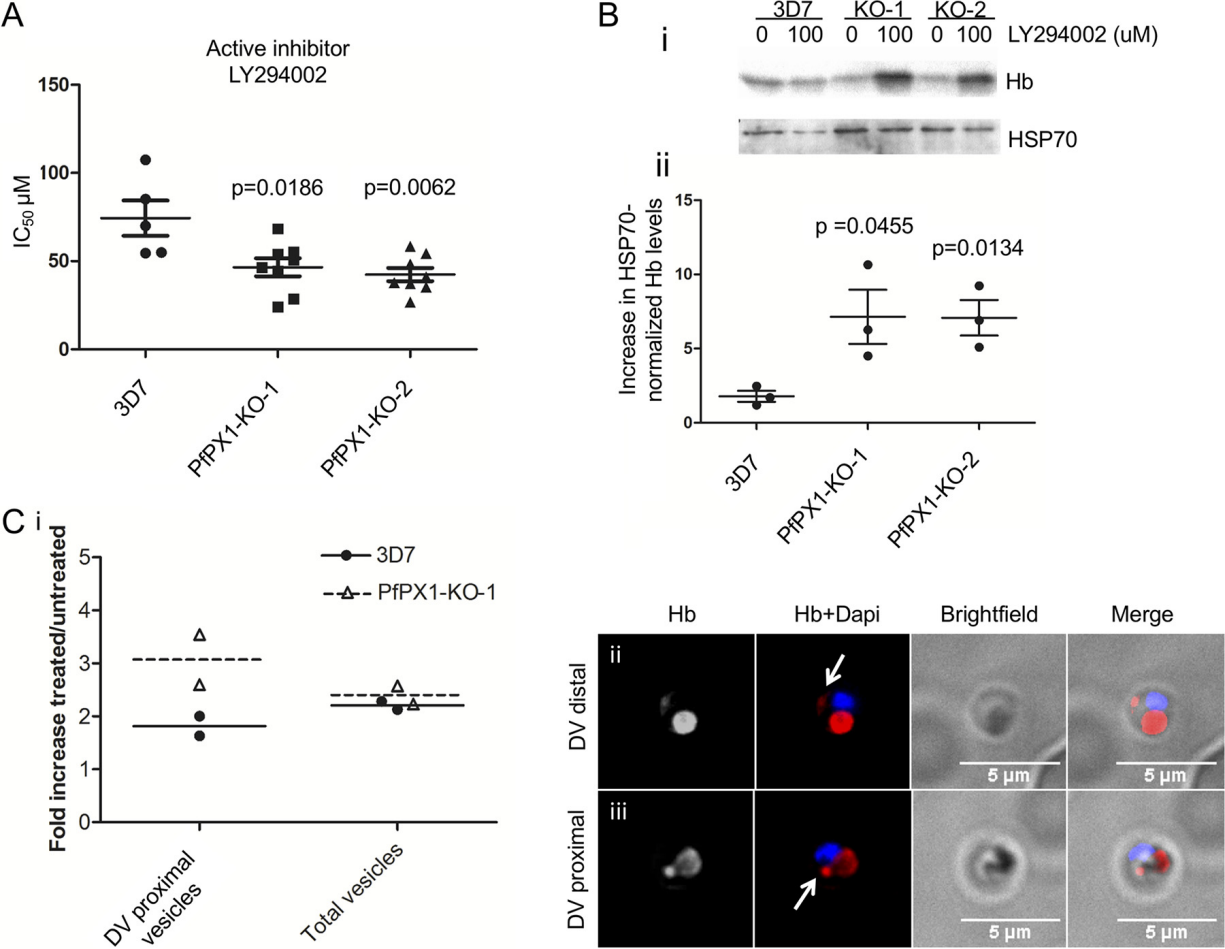
PfPX1 knockout lines are more sensitive to PI3K inhibition. (A) *In vitro* drug sensitivity assays with the PI3K inhibitor LY204002 were set up using synchronous ring stage parasites and % growth was measured 72 h later. Shown are the average IC_50_ values from at least 3 independent biological replicates. A two tailed paired *t* test was performed for statistical comparison. (B, i) Western blot from a representative experiment showing the effect of LY294002 treatment on parasite-associated Hb levels. Trophozoite (3D7) and ring/trophozoite (PfPX1-KOs) stages were treated with 100 μM LY294002 for 4 h. HSP70 was used as a loading control for normalization. Results from one experiment representative of 3 independent biological replicates shown. (B, ii) Quantification of the increase in parasite-associated Hb normalized to HSP70 upon LY294002 treatment from Western blot by densitometry analyses of three independent biological replicates. Unpaired *t* tests were performed for statistical evaluation. (C, i) Quantification of the increase in DV proximal and total Hb-containing vesicles upon LY294002 treatment. IFA using an anti-Hb antibody was performed on parasite lines at trophozoite (3D7) and ring/trophozoite (PfPX1-KO-1) stages treated with 100 μM LY294002 for 4 h. Mean ± SD from 2 independent biological replicates is shown. (C, ii) Representative image of a DV distal vesicle. (C, iii) Representative image of a DV proximal vesicle. White arrows: Highlighting the Hb vesicles. Dapi: parasite nuclei.

The colocalization of PfPX1 with HCC and Hb vesicles and with proteins involved in HCC endocytosis led us to hypothesize that PfPX1 could perhaps also play a role in the trafficking of HCC/Hb from the host cell to the DV. To investigate this, we first quantified the amount of Hb associated with the parasites by Western blot using an antibody against Hb. To specifically quantify parasite-associated Hb, the infected RBCs were treated with saponin to release the HCC. Due to the heterogeneity of the PfPX1-KO growth, the interpretation of the phenotype is less straightforward than directly comparing 3D7 and the KOs at a set time point. At the 24–30 hpi time point, when the 3D7 parasites are early trophozoites, the KO lines have more rings (thus less trophozoites) and around half of the KO parasites will be dead pyknotics. To increase the proportion of KO parasites that had reached the trophozoite stage and thus facilitate the interpretation of the data, we monitored their development until at least 50% had reached the trophozoite stage before harvesting. This meant a delay of 4–6 h between the harvest of 3D7 WT and of the KO parasites (e.g., 3D7 at 24–30 hpi and the KOs at 28–34 hpi). Despite this delay in harvesting, the KO populations still had a proportion of rings. Thus, in the assays looking at whole parasite populations like Western blots (Fig. 6A and 7B) and heme fractionation assays (Fig. 6B), the data obtained for the KO lines comes from both rings and trophozoites (first time point) and trophozoites and schizonts (second time point, for Hb Westerns only). This means that the Hb levels we detect in the KO population at this time point come from around half the number of viable cells, at least half of which are trophozoites, compared to WT. Intriguingly, whilst the levels of Hb (normalized to HSP70) in two independent PfPX1-KO clonal lines were higher than in the 3D7 control at the first time point (rings/trophozoites; PfPX1-KO-1 versus 3D7: 6.1-fold, PfPX1-KO-2 versus 3D7: 4.8-fold), this was no longer the case with more mature forms (trophozoites/schizonts) (Fig. 6A, Appendix Fig. 5). It is well established that HCC endocytosis is most prominent in the later erythrocytic stage parasites and therefore that ring stage parasites have endocytosed less Hb than trophozoites (40, 42, 46). As we can detect an increase in Hb in the KOs despite them having less trophozoites than 3D7 WT, this suggests that there is an important defect either in the trafficking pathway of Hb after the initial endocytosis step or with its degradation in the DV.

This increase in Hb content could potentially be due to either a defect in DV function resulting in a decrease in Hb digestion or it could be due to a decrease in the delivery of Hb-containing vesicles to the DV. To determine where in the Hb trafficking and digestion pathway PfPX1 potentially plays a role, we performed heme fractionation assays on 3D7 (trophozoites) and PfPX1-KO parasites (ring/trophozoites) to determine the proportion of heme found as cytosolic Hb, DV-associated free heme/Hb and hemozoin. For both PfPX1-KO lines, the portion of the total amount of cellular heme made up of cytosolic Hb was increased compared to the 3D7 control (PfPX1-KO-1 versus 3D7: 1.5-fold, PfPX1-KO-2 versus 3D7: 2.4-fold) (Fig. 6Bi). However, the amount of free heme/Hb in the DV was decreased in both KO lines compared to 3D7 (PfPX1-KO-1 versus 3D7: 1.9-fold, PfPX1-KO-2 versus 3D7: 1.4-fold) (Fig. 6Bii). Finally, the percentage of total cellular iron present in the hemozoin fraction was similar between WT and PfPX1-KO-2 (3D7: 94.6±0.4, PfPX1-KO-2: 95.0±0.5) but there was a small but statistically significant difference between WT and PfPX1-KO-1 (3D7: 94.6±0.4, PfPX1-KO-1: 96.5±0.1) potentially due to the very tight values obtained between the different bioreplicates (Fig. 6Biii). These data suggest that the absence of PfPX1 does not compromise the function of the DV, at least with regards to Hb digestion and likely subsequent hemozoin polymerization, and that there is indeed a defect in the delivery of Hb vesicles to the DV. To more directly assess Hb delivery to the DV, we performed DV bloating assays as previously described (79). Here, incubation of parasites with the protease inhibitor E64 for several hours prevents the degradation of newly delivered Hb in the DV and results in the swelling of the organelle. This swelling is not/less observed in parasites where Hb delivery to the DV is compromised as seen when PfVPS45 is inactivated for example (21). We thus incubated 3D7 WT (trophozoites) and PfPX1-KO-1 (ring/trophozoites) parasites for 8 h with E64 and measured their cellular and DV diameters. This revealed that in absence of PfPX1, incubation with E64 leads to reduced swelling of the DV compared to 3D7 WT which further supports that the parasites are defective in the delivery of Hb to the DV (ratio DV/cell diameter: 3D7: 64.2%±1.5, PfPX1-KO-1: 39.5%±1.8) (Fig. 6C). Importantly, this decreased swelling is not due to the fact that the PfPX1-KO-1 trophozoites are younger since there was no difference in their cellular diameter compared to the 3D7 control (3D7: 4.57 μm±0.13, PfPX1-KO-1: 4.68 μm±0.14) (Fig. S9). It is important to keep in mind that because of the heterogeneity of the KO population, a portion of the parasites had not reached the trophozoite stage and therefore did not have DVs so these were not taken into account. The results obtained thus show that even the parasites that seemed to have morphologically properly developed to the trophozoite stage potentially also had some defects in the delivery of Hb to the DV.

10.1128/mbio.03239-21.9 FIG S9 Digestive vacuole bloating assay. Data associated with Fig. 6C. Diameter of the digestive vacuole and of the parasite after an 8-hour incubation with E64 for 3D7 and PfPX1-KO-1 lines. Each data point represents one cell. Values from 3 independent biological replicates are pooled together. A minimum of 20 cells per replicates were measured. N: 62 for 3D7 and 65 for PfPX1-KO-1. A two-tailed unpaired *t* test was performed for statistical analysis comparing cell diameters and DV diameters. DV: digestive vacuole. Download FIG S9, PDF file, 0.1 MB. Copyright © 2022 Mukherjee et al. 2022 Mukherjee et al. https://creativecommons.org/licenses/by/4.0/ This content is distributed under the terms of the Creative Commons Attribution 4.0 International license.

We next reasoned that a defect in Hb delivery to the DV should result in an increase in the number of Hb vesicles inside the parasite cytoplasm. We thus performed IFA to look at the number of Hb-containing vesicles (assuming that the Hb-containing foci observed represent vesicles) in 3D7 WT (trophozoites) and PfPX1-KO-1 (ring/trophozoites) parasites and saw a significant decrease in the number of the PfPX1-KO-1 parasites with no vesicles compared to 3D7 (3D7: 29.3±0.3, PfPX1-KO1: 21±1) and a significant increase in the number of parasites containing two vesicles in the PfPX1-KO-1 strain compared with 3D7 (3D7: 5.7±0.7, PfPX1-KO-1: 10.7±2.2) (Fig. 6D, Appendix Fig. 5). However, no difference was seen in the number of parasites with 1 vesicle. One or two parasites with three or four vesicles were also observed in both parasite lines (Fig. 6D). In conclusion, the data suggest that the increase in parasite-associated Hb observed in absence of PfPX1 is not due to a default in DV function but potentially to a defect in the delivery of Hb-containing vesicles to the organelle.

### Absence of PfPX1 results in a decreased ability to grow in an amino acid-limited medium.

P. falciparum is auxotrophic for most amino acids and obtains most of them through digestion of host Hb by proteases present in the DV (80). Indeed, during the P. falciparum asexual cycle, up to 80% of the host cell cytosol is ingested and trafficked to the DV (81). To increase the parasite’s reliance on Hb degradation for its nutritional needs, a medium containing only five amino acids that are absent or poorly represented in Hb (cysteine, glutamate, glutamine, methionine and isoleucine) was previously shown to affect parasite growth (80, 82, 83). We reasoned that the defect in Hb trafficking to the DV in the KO lines would result in them growing less efficiently in this amino acid-restricted medium. Indeed, while WT growth in AA-restricted medium is 47% of its growth in complete medium; that is, it grows 53% less, the KOs had growths in AA-restricted medium of 28% (PfPX1-KO-1) and 31% (PfPX1-KO-2) of their growth levels in complete medium which means a decrease of 72% and 69% respectively (Fig. 6E). These data provide further evidence that the delivery of Hb from the host cell cytosol to the DV is compromised in the PfPX1-KO lines.

### The PfPX1 KO parasites are hypersusceptible to PI3K inhibition.

Treatment of P. falciparum parasites with the PI3K inhibitors Wortmannin and LY294002 results in increased Hb levels and increased Hb-containing vesicles and this led to the proposal that PfPI3K was involved in the endocytosis of host Hb and its subsequent trafficking to the DV^47^. These data resemble our phenotype of the PfPX1-KOs which further hinted that PfPX1 was involved in the same process. We therefore hypothesized that the PfPX1-KO parasites would be more sensitive to PI3K inhibition due to an exacerbated increase in parasite-associated Hb and potentially Hb-containing vesicles. To test this, we first determined the IC_50_ of 3D7 and the two PfPX1-KO lines to LY294002 by standard *in vitro* drug susceptibility assays. As a control, we used LY303511 a derivative of LY294002 inactive against PI3K^70^. We observed that both PfPX1-KO lines were indeed more susceptible to PI3K inhibition (Fig. 7A: 3D7: 74±10 μM, PfPX1-KO-1: 46±5 μM, PfPX1-KO-2: 42±4 μM) but not to the inactive compound (Fig. S10 in the supplemental material).

10.1128/mbio.03239-21.10 FIG S10 Sensitivity of the parasite lines to LY303511, an inhibitor inactive against PI3K. A two tailed paired *t* test was performed for statistical comparison. N = a minimum of 3 independent biological replicates. Download FIG S10, PDF file, 0.1 MB. Copyright © 2022 Mukherjee et al. 2022 Mukherjee et al. https://creativecommons.org/licenses/by/4.0/ This content is distributed under the terms of the Creative Commons Attribution 4.0 International license.

To test if the hypersusceptibility of the PfPX1-KO lines to PI3K inhibition was due to exacerbated Hb accumulation, we exposed 3D7 WT (trophozoites) and PfPX1-KOs (ring/trophozoites) parasites to 100 μM LY294002 for 4 h and quantified the parasite-associated Hb levels by Western blot. This revealed that LY294002-treated PfPX1-KO-1 and KO-2 parasites accumulated 4-fold higher levels of Hb than the 3D7 control line (Fig. 7B, Appendix Fig. 6). To assess if the increased Hb levels were due to higher accumulation of Hb-containing vesicles in the PfPX1-inactivated lines, we quantified the number of Hb-containing vesicles by IFA in PfPX1-KO-1 rings/trophozoites and 3D7 trophozoites pretreated with 100 μM LY294002 for 4 h. Interestingly, while both parasite lines had an increase in DV-proximal vesicles caused by PI3K inhibition, this increase was almost twice as much in the KO (Fig. 7C). However, there was no major difference in the total number of Hb vesicles (Fig. 7C). As previously demonstrated, Hb vesicles and the DV membrane are labeled with PI3P and treatment of parasites with PI3K inhibitors reduces PI3P levels which likely leads to a less efficient fusion of Hb vesicles (17, 20, 21, 47). This could result in more Hb vesicles proximal to the DV membrane. We speculate that this is potentially exacerbated in the PfPX1-KO-1 line due to the combination of the absence of PfPX1 and the reduction in PI3P which could explain why we see more DV proximal Hb vesicles.

### Inactivation of PfPX1 reduces ART susceptibility as determined by Ring Stage Survival Assay.

**FIG 8:**
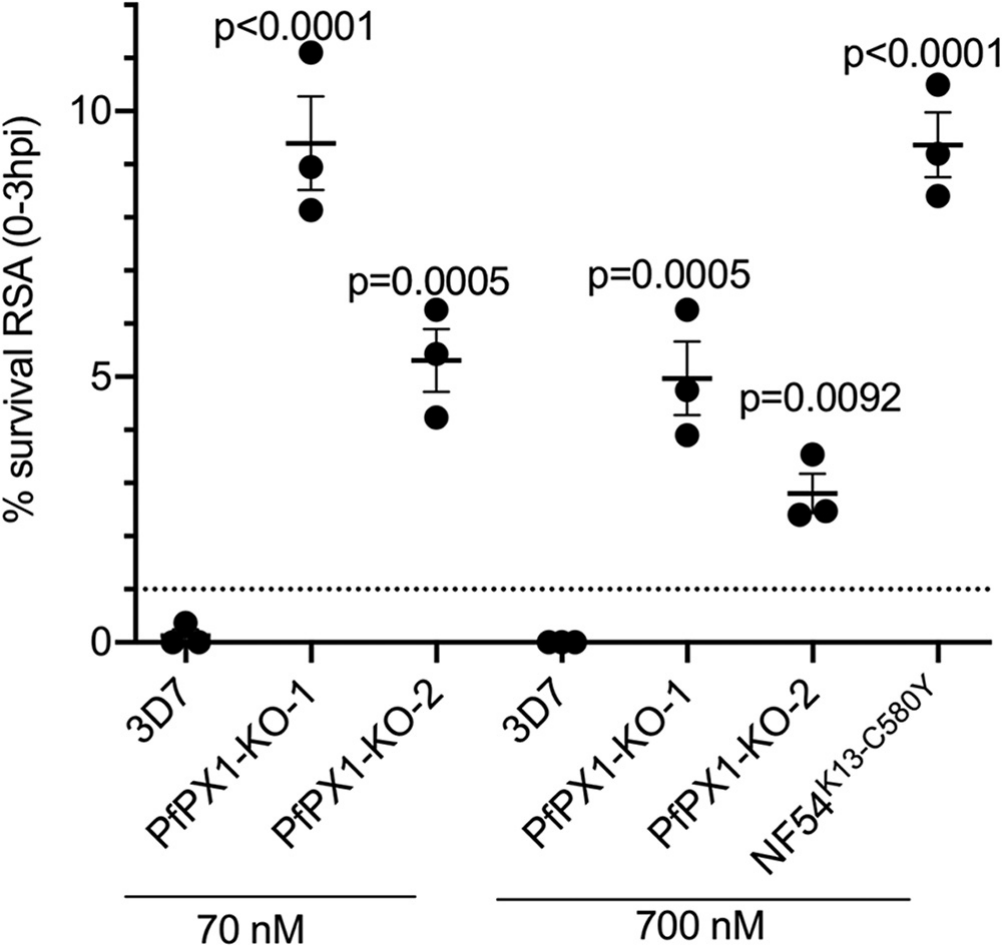
PfPX1 knockout lines have reduced susceptibility to DHA by RSA. RSA_0-3h_ was performed starting with 0 to 3 h post invasion rings of 3D7 and two independent PfPX1-KO lines with 70 and 700 nM DHA. NF54^K13C80Y^ engineered line was used as a positive control for 700 nM DHA treatment. Percentage of survival was calculated as percentage of next generation healthy-looking DHA-exposed parasites to controls. Results are mean ± SEM of 3 biological replicates. One-way ANOVA with Sidak’s multiple comparison test was performed. Dotted line at 1% survival shows the cut off used to discriminate between sensitive and resistant lines.

Hb trafficking and metabolism are central to the mechanisms of ART action and resistance (46, 84–87). Moreover, recent work has shown that PfPI3K is a target of ARTs (88, 89). To characterize the effects of dihydroartemisinin (DHA) in the PfPX1-KO lines, we performed a standard ring survival assay (RSA) (81, 82). 0-3 h post invasion (hpi) rings were exposed to 70 and 700 nM DHA and 0.1% DMSO (control vehicle) for 6 h, washed and incubated in drug free medium. Parasite survival was quantified in the next cycle, 66 h later. In this assay, natural and genetically engineered parasite strains with RSA survival rates >1% are considered resistant to DHA (44, 45, 90). As a positive control for the 700 nM DHA treatment, we used the NF54^K13-C580Y^ parasite line where the marker for artemisinin resistance K13 was genetically engineered to K13C580Y to generate *in vitro* artemisinin resistance in the lab strain NF54 (91). This parasite line is now used as the gold standard in RSAs (72, 91). As expected 3D7 had <1% survival at both 70 and 700 nM DHA treatment whilst NF54^K13-C580Y^ demonstrated a survival of 9.4±0.61% (91) at 700 nM. In our PfPX1-KO lines, we observed an increased survival to DHA exposure compared to 3D7 for both concentrations (Fig. 8; 70 nM: 3D7: 0.12±0.12%; PfPX1 KO-1: 9.4±0.9%; KO-2: 5.3±0.6%. 700 nM: 3D7: 0%; PfPX1 KO-1: 5.0±0.7%; KO-2: 2.8±0.4%). Globally, these data demonstrate that the absence of PfPX1 results in a reduction in ART sensitivity in ring stages which could potentially be explained by a default in the Hb trafficking pathway.

## DISCUSSION

Phosphoinositides (PPIs) clearly play crucial roles in several aspects of the biology of malaria parasites and notably, one of the parasite’s PI4-kinase is now the target of an inhibitor currently in development as an antimalarial (24, 92). This validation of PPI metabolism as a source of potential antimalarials demonstrates the importance to develop a more comprehensive understanding of the impact of these lipids in the biology of the parasite. The localization of PI3P on the DV membrane and on intracellular vesicles and the disruption of Hb trafficking to the DV upon inhibition of PfPI3K argue for a role for PPIs in the process. To shed further light on this, we attempted to inactivate genes coding for proteins putatively involved in PPI metabolism. We demonstrate that 79% of identified genes could not be inactivated and are therefore potentially essential for *in vitro* asexual growth, speaking to the importance of PPIs in *Plasmodium*. The gene with the most severe KO phenotype codes for a PX-domain containing protein, which we show to be *Plasmodium*-specific and potentially involved in the Hb trafficking pathway based on several lines of evidence.

Our inability to generate KOs for a majority of the putative PPI candidates suggests that there is potentially minimal redundancy in this pathway as previously proposed (16, 22). When comparing our data with the genome-wide saturation P. falciparum Piggyback transposon mutagenesis screen (57), we see concordance for four out of five genes that we inactivated (Table 1). The putative PPI phosphatase PF3D7_1111600 has a mutagenesis score of 0.12 which suggested that it was essential. However, our ability to inactivate it suggests otherwise, at least in the 3D7 strain that is used in our laboratory. For the genes that we couldn’t knock out, three of them have mutagenesis scores of 1 or close to 1, suggesting that they were not essential. Two of these code for potential PPI phosphatases. We had earlier shown that knocking down PfSAC1 expression by more than 95% did not impact asexual growth (93) so perhaps our inability to inactivate it using SLI is due to technical difficulties and not essentiality. It is interesting to note that of the four putative PPI phosphatases in our screen, three of them had discordant results with the transposon screen. We have no explanation at this stage for this discrepancy. Quite good concordance was seen with the genes that were conserved in T. gondii and their fitness score based on the whole genome CRISPR screen (94) (16 genes total, 3 discordant). On the other hand, half of our candidate genes (12/24) did not have data in the P. berghei whole genome KO screen and we find discordance in half of those that are present (6/12) (95). Of interest, most of the P. berghei homologues of the P. falciparum genes are not essential for asexual proliferation in the whole genome Pb screen (8/12). However, several of them were shown to be important in the transition from the blood stage to midgut oocysts (96).

A role for PfPI3K and its product PI3P in Hb trafficking was proposed previously (17, 47, 48) but what this role would be and the identity of PPI binding effectors involved remained elusive. Our work has identified a PX domain-containing protein that likely binds PI3P. Our evidence that PfPX1 is potentially important in the trafficking pathway of Hb to the DV is based on several lines: i) the protein localizes to vesicular structures and the DV membrane. Some of these vesicles/foci contain HCC/Hb and some colocalize with known markers of HCC endocytosis; ii) the PfPX1-KO lines have increased levels of cytosolic Hb and potentially Hb-containing vesicles; iii) results from the DV bloating assay suggest that delivery of Hb to the DV is compromised in the PfPX1-KO mutants; iv4) the PfPX1-KO parasites have a decreased ability to grow in an amino-acid limited medium that increases their reliance on Hb digestion (and therefore on its delivery to the DV); v) the PfPX1-KO lines are more sensitive to PI3K inhibition and 6) the PfPX1-KO lines have reduced susceptibility to DHA. Importantly, the results from the heme fractionation assays suggest that DV function and more specifically Hb digestion and likely hemozoin polymerization, is not compromised in the PfPX1-KO lines. We speculate that the interaction of the PX domain of PfPX1 with PI3P perhaps facilitates the tethering of the Hb-containing vesicle and that this leads to its more efficient fusion to the DV membrane. However, our data does not allow us to discriminate whether PfPX1 or PI3P is on the DV membrane or on the Hb-containing vesicle. In absence of PfPX1, it is possible that fusion between Hb-containing vesicles and the DV could still occur, albeit less successfully. This stochastic colliding of Hb-containing vesicles to the DV membrane could therefore perhaps explain the heterogeneity in the phenotypic consequences observed in the PfPX1-KO strain: dead pyknotic parasites, delayed growth and normal progression.

The function played by the rest of the protein is unknown at this stage since no obvious domain can be identified apart from a large intrinsically disordered region. The flexibility of disordered domains in transmembrane proteins is important in various processes such as the tethering of signaling complexes (97, 98) and in regulating the function of channels and transporters (99). Interestingly, disordered regions can also act as drivers of membrane interactions such as membrane fusion and curvature (100, 101). Our topology analysis shows that the C-terminus of PfPX1 faces the cytosol and it is likely the same for the N-terminal PX domain if it is to interact with PI3P on membranes facing the cytosol. Based on the prediction that PfPX1 possesses 6 putative TMs, this means that the disordered region would be intraluminal where it could therefore not interact with intracytosolic factors. Perhaps the disordered domain interacts with proteins inside the vesicle/DV to help with packaging/release. In absence of further evidence, this should be considered pure speculation.

Our ability to inactivate the gene coding for PfPX1 clearly shows that it is not essential for *in vitro* growth though the severe growth defect of the KO strain indicates that it is important. Indeed, for all the different types of assays we performed, the phenotypes observed were never as striking as what was seen with essential factors such as PfVPS45 whose functional inactivation by knock-sideways led to complete parasite death. In this case, the accumulation of HCC-filled vesicles was at least five times more than what we saw with the PfPX1-KO^21^. Whilst both proteins could potentially be implicated in the maturation and/or fusion of Hb-containing vesicles to the DV membrane, the parasite stage where their phenotypes occur differ. PfVPS45 mutants arrest at the trophozoite and schizont stages whilst the PfPX1-KO lines potentially falter somewhere during ring and early trophozoites. The heterogeneity of the parasite population of the PfPX1-KO strains (pyknotics, delayed, normal) makes it hard to pinpoint exactly where the phenotype occurs with more confidence however the results from the DV bloating and Hb fractionation assays show that in absence of PfPX1, delivery of Hb to the DV is compromised, like in the PfVPS45 mutants. What is clear is that both PfPX1 and PfVPS45 act at steps both subsequent to the recently described early endocytosis phase involving PfK13, PfAP2μ and other proteins where conditional inactivation did not lead to an accumulation of HCC-filled vesicles (21, 42, 46).

The process of membrane fusion is based on a series of steps largely conserved in eukaryotic cells (102). Initial priming of the membrane of the vesicle is followed by its tethering to the target organelle, after which the formation of trans-SNAP receptor (SNARE) complexes results in the docking of the vesicle and the formation of the vertex ring, the zone at the edge of the circular point of contact between the two membranes and where the lipids and proteins regulating fusion become enriched(reviewed in (103–106)). While in the absence of a tether vesicles bearing SNAREs can sometimes spontaneously form trans-SNARE complexes and occasionally trigger fusion, multisubunit tethering complexes (MTC) facilitate the formation of fusion competent trans-SNAREs and potentially lower the energy barrier required for fusion (103, 107). The CORVET complex serves as the MTC at early endosomes, with the HOPS complex performing the same function at late endosomes and lysosomes/vacuole (108). These complexes share a four proteins core (the VpsC core), but differ in their peripheral Rab-interacting proteins, Vps3/8 (Rab 5 interactors) and Vps39/41 (Rab 7 interactors) for CORVET and HOPS respectively. Notably, while *Plasmodium* and all apicomplexans possess homologues for the VpsC core, *Plasmodium* lacks the HOPS-specific subunits, and Vps8 of the CORVET complex (109, 110). Given that HOPS functions in delivery of vesicles at the lysosome, and based on our data that the *Plasmodium*-specific PfPX1 potentially serves an analogous function, we speculate that the PfPX1 evolved in the *Plasmodium* lineage and provided a selective advantage to enhance Hb-containing vesicle delivery to the digestive vacuole. Evolution of lineage-specific tethering factors involving PPI binding has precedent. In yeast, the Qc-SNARE Vam7p possesses a PX domain which promotes the tethering of vesicles by its trans-interaction with PI3P on the surface of the vacuole (111, 112). Notably, however, this PX domain is a lineage-specific feature of Fungi and not universal to Vam7 orthologues (Syntaxin 8) in other eukaryotes (113, 114). Intriguingly, the PX domain of Vam7p is required only to target the protein to PI3P containing membranes and a mutant with abrogated binding can still support membrane fusion albeit at much higher concentrations of protein since the colliding of membranes bearing SNAREs can lead to the spontaneous formation of trans-SNARE complexes and this can occasionally result in fusion (107). Perhaps a similar situation occurs in the PfPX1-KO.

In conclusion, our KO attempts of putative PPI-related proteins highlighted the potential minimal redundancy of the PPI pathway in P. falciparum and led to the identification of a *Plasmodium*-specific PI3P-binding protein potentially implicated in the trafficking pathway of Hb to the DV. The essentiality of this process for malaria parasite survival and its recent implication in ART resistance demonstrate the importance of developing a better understanding of the underlying mechanisms. Finally, the characterization of the other newly generated KO lines will further advance our comprehension of PPI metabolism in P. falciparum, a validated target for the development of antimalarial drugs.

## MATERIALS AND METHODS

The study was approved by the Canadian Blood Services (CBS) research ethics board, project number 2015.001 and by the CHU de Québec IRB, project number 2015–2230, B14-12-2230, SIRUL 104595. Written consent was obtained by the CBS for all study participants. All experiments were performed in accordance with relevant guidelines and regulations.

### Parasite culture.

P. falciparum 3D7 asexual stage parasites (from David Walliker, Edinburgh University) were cultured under standard conditions in RPMI-HEPES medium at 4% hematocrit (human erythrocytes of O^+^ group) and 0.5% (w/v) Albumax^TM^ (Invitrogen) and kept at 37°C in a gas mixture of 5.0% oxygen, 5.0% carbon dioxide and 90% nitrogen (115). For growth assays in amino acid-limited medium, RPMI-HEPES lacking all amino acids was made to which isoleucine, glutamine, glutamate, methionine and cysteine (Sigma) were added at the concentration found in regular RPMI.

### Vector construction and transfection.

To disrupt the genes of interest (GOI) using the recently described SLI-TGD, around 500 bp of the N-terminus of the GOI was amplified with primers containing 5’Not1 and 3’Mlu1, digested and cloned in frame with GFP in pSLI-TGD digested NotI-Mlu1^56^. To prevent expression from the episome, the start codon ATG was replaced by a stop codon TAA in the 5’ primer. Primers listed in Table S1. To detect the 5’ integration event, a GOI specific forward primer along with primer GFP85-rev (ACCTTCACCCTCTCCACTGAC) were used. For the 3’ integration event, primer pArl-fw (GGAATTGTGAGCGGATAACAATTTCACACAGG) and a GOI specific reverse primer were used. Finally, to detect the WT allele, the GOI specific forward and reverse primers were used. Primer sequences listed in Table S2 in the supplemental material.

To tag the endogenous PfPX1 with GFP, around 350 bp of the C-terminus of PfPX1 was amplified with primers 5'NotI-6999-PfPX (ATAGCGGCCGCTTTTAGGTGGGACGATAAAATC) and 3'MluI-stopless-PfPX (ATAACGCGTAAAAAGTTGACAATCATTTTCATC) and cloned in frame with GFP in pSLI-GFP digested NotI-AvrII^56^. To tag the endogenous PfPX1 with smHA, the tag was amplified on plasmid pJR118 (116) using 5’ MluI-atgless-smHA (ATACGCGTTACCCTTATGATGTGCCCGA) and 3’revSalI-stopless-smHA (TAGTCGACAGCGTAGTCCGGGACATCGTAC), digested and cloned in place of GFP in pSLI-PfPX1-GFP digested Mlu1-Sal1.

Parasites were transfected and integrants were selected as described previously with some modifications (56). Briefly, P. falciparum 3D7 parasites were transfected with 100 μg of pSLI plasmids. Positive selection for transfectants was achieved using 5 nM WR99210 (WR, Jacobus Pharmaceuticals). Then drug resistant parasites were split into 3 separate wells with 1–2% parasitemia and went under another round of selection using 400 μg/ml neomycin (NEO) to select for integrants. After parasite reemergence (after around 10 days) WR was put back in the culture medium. Parasites were than cloned by limiting dilution. Genomic DNA was prepared from NEO and WR resistant parasites. Integration was monitored by PCR using the forward 5'upstream-GOI-F (primer 1) and the reverse 3'90-GFP-R primer (primer 2) for 5' integration and 5'pARL-F (primer 3) with the reverse 3’-3UTR-GOI-R primers (primer 4) for the 3' integration. Primer 1 was used with primer 4 to detect the WT version of the gene. (see Table S2 for primer sequences).

To generate the pLN-PXdom-GFP vector, the PX domain of PfPX1 was amplified with primers 5'AvrII-PfPX (ATACCTAGGATGATGGAAAGTTATTCAACATATGAATTTAAAG) and 3'XhoI-390-PfPX (ATACTCGAGTTTTGTTATATTATCTAAGCAC) and cloned in phase with GFP in pLN-ENR-GFP-BSD (117) digested AvrII-XhoI, Parasites were transfected and selected with 200 μg/ml blasticidin S-HCL (BSD) (Wisent).

To generate the pHSP86p-VPS45-mScarlet-DHODh and AP2μ-mScarlet, the coding sequence for each gene was amplified by PCR from 3D7 cDNA using 5’AvrII-VPS45 (ATACCTAGGATGGAGAATAATCCTTACGTG) and 3’Kpn1-stoplessVPS45 (ATAGGTACCTTTCTTGATAAGCTGCAAAAC) for PfRab5a and 5’AvrII- AP2μ (ATACCTAGGATGATCGATGCGCTGTACATATTTTTTATTAACG) and 3’Kpn1-stopless AP2μ (ATAGGTACCTTTATACTGGTAGATGCCCGATTC).

To generate the pHSP86p-mScarlet-Rab5a-DHODh and Rab7 plasmids, the coding sequence for each gene was amplified by PCR from 3D7 cDNA using 5’Mlu1-Rab5a (ATAACGCGTATGGAAAAGAAAAGTAGTTATAAAAC) and 3’Xho1-Rab5a (ATACTCGAGTTAACAACATCCTTTTTTTGAAAGTG) for PfRab5a and 5’Mlu1-Rab7 (ATAACGCGTATGTCAAATAAAAAAAGAACCATATTAAAAG) and 3’Xho1-Rab7 (ATACTCGAGTTAACAACAACGACTTTTGTACATTTTTTG).

To generate the double transfectants, the PfPX1-GFP line was transfected with 100 μg of each of the 4 pHSP86p-mScarlet-DHODh plasmids. Parasites were cultured with 5 nM WR and 0.9 μM DSM1 (BEI Resources).

### Mammalian cell transfection and fluorescence confocal imaging.

EGFP-tagged PfPX domain expressing plasmids were generated by Gene Universal (Newark, DE, USA) using a pEGFP-N1 vector. p40^phox^-EGFP (14), mCherry-Rab9a (118), mCherry-Rab5, mCherry-Rab11 and mCherry-LAMP1 were gifts from Michael Davidson. All plasmids were provided from Addgene (#19010, #78592, #55126, #55124, #55073, respectively). Cos-7 cells were co-transfected with EGFP-PX and mCherry expressing plasmids using lipofectamine 2000 reagent according to the manufacturer’s instructions (Invitrogen). At 18-20 h post-transfection, cells were stained for DNA and plasma membrane, Hoechst3334 and wheat germ agglutinin-AlexaFluor647 (Molecular Probes^TM^), respectively for 15 min at 37°C, fixed with 4% paraformaldehyde (PFA) for 15 min at room temperature then washed twice with PBS. For treatment with kinase inhibitors, cells were treated with vehicles, 100 nM Wortmannin or 20 nM Apilimod for 1 h at 37°C, 18-20 h post-transfection. Cells were then stained for DNA and plasma membrane as described above. Confocal images of transfected cells were captured using a Nikon Eclipse Ti Confocal inverted microscope (Nikon, Japan), using a 100x 1.45 numerical aperture oil objective, and cells were imaged using the 405 nm, 488 nm, 594 nm and 647 nm argon lasers to excite Hoechst, EGFP, mCherry and WGA-Alexa Fluor 647, respectively. Histogram analysis was performed using ImageJ (119) on regions of interest defined using the mCherry channel on a total of 37-60 cellular punctae from three independent replicates.

To determine whether the localization of the PfPX1 PX domain was dependent on PI3P, pLN-PXdom transfected parasites at 28-34 hpi were treated with 250 μM of the PI3K inhibitor LY294002 (IC90) and 50 μM of its inactive equivalent LY303511 (IC90) as control for 4 h. 0.25% DMSO was used as second control as it’s the highest concentration used in the experiment (to reach 250 μM LY294002).

### Homology searching and informatic analyses.

PfPX1 (PF3D7_0720700) was used as a query for searches using BLAST (120) into the protein databases of *A. castellani, A. thaliana,* B. microti*, B. natans, C. cayentanensis,* C. parvum*, C. suis,* C. velia, D. discoideum*, E. necatrix,* G. theta*, H. hammondi,* H. sapiens, N. caninum*, P. adleri,* P. berghei*, P. billcollinsi, P. blacklocki,* P. chabaudi, P. coatneyi, P. cynomolgi, P. falciparum, P. fragile*, P. gaboni, P. gallinaceum, P. inui,* P. knowlesi*, P. malariae, P. marinus, P. ovale curtisi, P. praefalciparum, P. reichenowi, P. relictum, P. sojae,* P. vivax*, P. yoelli,* S. cerevisiae*, S. minutum, T. annulata,* T. brucei, T. gondii, T. thermophila, T. vaginalis and V. brassicaformis, using in‐house scripts. Potential orthologues were then used as queries for a homology search using BLAST into the P. falciparum protein database. Candidates were considered positive orthologues when both forward and reverse BLAST hits generated an E‐value below a 0.05 threshold, if the candidate protein in question retrieved the relevant query with e‐values two orders of magnitude better compared to the next non‐redundant hit, and if the top reverse BLAST hit was PF3D7_0720700. HMMer (v3.2.1) (121), searches were also conducted using a HMMer profile made of PF3D7_0720700 known orthologues into the protein databases of the organisms listed above. The HMMer profile was generated using MUSCLE alignment software (v3.8.31) (122), and Fetch software (v5.7.7 downloaded from https://fetchsoftworks.com) was used to search into the protein databases. Potential orthologues were validated using the above procedure. BLAST and HMMer searches were performed for both the full‐length PF3D7_0720700 query and PF3D7_0720700 without the PX domain (amino acids 16–125). In the case where searches were performed without the PX domain, CDD on NCBI (https://www.ncbi.nlm.nih.gov/Structure/cdd/wrpsb.cgi) identified the location of the PX domain and Emboss: extractseq (http://www.bioinformatics.nl/cgi-bin/emboss/extractseq) was used to trim the PX domain from the query. Conserved domain structures of the orthologues were identified using Phyre v2.0 (http://www.sbg.bio.ic.ac.uk/phyre2/html/page.cgi?id=index).

### Western blotting.

For the time-course of expression analysis, parasites were synchronized twice at an 18-h interval (resulting in 18 and 22 h ring stages), with a 0.3 M alanine-10mM HEPES solution (as described in (123)). Synchronous parasites were then harvested by saponin lysis, the pellets solubilized in SDS protein sample buffer and separated on a 7.5% SDS-PAGE gel under reducing conditions and transferred to PVDF membranes (Millipore). The antibodies rabbit polyclonal anti-PfHSP70 (SPC-186C; StressMarq Bioscience Inc., 1:20000) (124), rabbit polyclonal anti-BiP (1:1000) (125), rabbit polyclonal anti-plasmepsin II (126) (1: 2000), mouse monoclonal anti-GFP (Roche, JL8, 1:1000) and mouse monoclonal anti-HA (Cedarlane, CLH104AP, 1:2000), were diluted in 0.1% (v/v) Tween 20-phosphate-buffered saline with 1% (w/v) skim milk. Appropriate HRP-coupled secondary antibodies were used and immunoblots were revealed by ECL (Amersham Biosciences). For all expression analyses, proteins extracted from an equal number of cells were used for each time point.

### Fluorescence imaging.

Fluorescence images of parasites were captured using a GE Applied Precision Deltavision Elite microscope with 100x 1.4NA objective and with a sCMOS camera and deconvolved with the SoftWorx software. Chromatic calibration of the microscope was performed prior to imaging experiments. For immunofluorescence assays, parasites were fixed on slides using 4% paraformaldehyde (ProSciTech) (127) and permeabilized with 0.1% Triton X-100 (Sigma-Aldrich). After blocking in 3% bovine serum albumin (Sigma Aldrich) the cells were incubated for 1 h with rabbit polyclonal anti-PfERD2 (1:2000) (128), mouse monoclonal anti-HA (Cedarlane, CLH104AP, 1:2000), goat anti-human Hb (1:1000, Cedarlane) or rabbit polyclonal anti-BiP (1:1000) (125). Bound antibodies were then visualized with either Alexa Fluor-594 anti-rabbit, anti-mouse or anti-goat IgG and Alexa Fluor-488 anti-mouse or anti-rabbit IgG diluted 1:1000 (Cedarlane). Parasites were mounted in Vectashield (Vecta Laboratories) containing 0.1 μg/ml 4', 6–diamidino-2-phenylindole (Dapi, Invitrogen). Images shown represent a single optical slice from a deconvolved z-stack. For the LysoTracker labeling experiment, PfPX1-GFP parasites were incubated for 2 h with 75 nM of LysoTracker Red DND-99 (Invitrogen).

### Preparation of red blood cell ghosts filled with dextran.

Red blood cells were preloaded with fluorescent dextran based on (21, 129). The RBCs were washed several times in cold PBS (Sigma-Aldrich) and 32 μl of the packed RBC pellet were recovered and carefully added to the preloading lysis buffer (freshly prepared using 64 μl of 5 mM K_2_HPO_4_/20 mM D-Glucose pH 7.4, 1 ml of 30 mM DTT, 2 ml of 50 mM MgATP and 1 ml (50 mg/ml) of Alexa Fluor 594-conjugated 10 kDa dextran (Thermo Fisher)). The resuspended RBCs in preloading lysis buffer were rotated at 4°C for 10 min and resealed by slowly adding 25 ml of the 5× preloading resealing buffer (750 mM NaCl/25 mM Na2HPO4 pH 7.4) into the lysis mixture and subsequent incubation at 37°C while gently rocking for 60 min. The pre-loaded cells were washed three times with RPMI and stored at 4°C until needed.

To infect the preloaded RBCs, PfPX1-GFP parasites were tightly synchronized by Percoll purification of late schizonts, followed 6 h later by a 5% D-sorbitol treatment. The parasites were then grown until they reached the schizont stage where they were again purified with Percoll and subsequently added to the dextran-filled RBCs to allow for reinvasion. Samples were taken at the ring, trophozoite and schizont stages and imaged by fluorescence microscopy.

### Proteinase K protection assay.

The Proteinase K protection assay was based on (130). 3x 10 ml of mixed-stage PfPX1-smHA parasites were lysed with saponin and the following treatments were performed: The first sample was resuspended in SOTE buffer alone (0.6 M sorbitol, 20 mM Tris HCl pH 7.5, 2 mM EDTA). The second sample was resuspended in SOTE with 0.02% w/v digitonin (Sigma-Aldrich), incubated for 10 min at 4°C and then washed with SOTE. The last sample was treated with digitonin followed by digestion with 0.1 mg/ml Proteinase K (Sigma-Aldrich) in SOTE for 30 min at 4°C. Proteinase K was inactivated by precipitation with 50 μl of 100% v/v trichloroacetic acid. The pellets were then washed in acetone and briefly dried before solubilization in protein sample buffer and subsequent analysis by Western blot.

### Hb-containing vesicles enumeration.

To determine the number of Hb containing vesicles/foci 3D7 WT trophozoites and PfPX1-KO rings/trophozoite were saponin-lysed, washed in PBS and immobilized on poly-L-lysine treated coverslips. The attached parasites were then fixed for 15 mins in PBS containing 4% (m/v) paraformaldehyde and 0.25% (m/v) glutaraldehyde, washed with PBS and then permeabilized with PBS-0.5% (v/v) Triton X-100 followed by a 20 min incubation in PBS-0.15M glycine to neutralize aldehydes. The usual IFA procedure was then followed using a goat anti-human Hb (1:1000, Cedarlane) and a donkey anti-goat IgG coupled to Alexafluor 594 (Cedarlane).

### Subcellular fractionation of PfPX1-smHA trophozoite-infected erythrocytes.

To analyze the processing of PfPX1 in the vesicular fraction, free from the digestive vacuole, parasites were ruptured by nitrogen cavitation and the vesicular fraction was isolated by differential centrifugation as previously described (131). Briefly, tightly synchronous PfPX1-smHA trophozoites were saponin-lysed, washed in PBS and resuspended in 1.7 ml cavitation buffer (10mM Hepes, 10mM KCl, 1mM EDTA, 250mM sucrose, pH 7.4) and 0.3 ml 7X protease inhibitor cocktail (Roche, 11836170001) and lysed in a nitrogen-pressure chamber at 49bar/800psi for 30 mins. The sample was then spun twice at 8000g for 10 mins at 4°C. The resulting pellet containing unbroken parasites, nuclei and digestive vacuoles and the supernatant containing the vesicular fraction were solubilized in SB and run on SDS page. Western blot was then performed using a mouse anti-HA (1: 2000, Cedarlane, CLH104AP) and a rabbit anti-plasmepsin II (126) (1: 2000).

### Hb fractionation.

The Hb fractionation assay was adapted from (132–134). Parasites were tightly synchronized by Percoll purification of late schizonts, followed 6 h later by a 5% D-sorbitol treatment. Cells were put back to the incubator for 4 h and then passed through a magnetized LS Column (Miltenyi) to remove dead parasites and free hemozoin coming from the ruptured schizonts. Parasites were further grown until they reached the trophozoite (3D7 WT) or ring/trophozoite (PfPX1-KO line) stages before being harvested and 100 x 10^6^ iRBC saponized with a 0.1% saponin solution in PBS with complete protease inhibitor cocktail (Roche) in triplicate. The parasites were washed three times with cold PBS and stored at −80°C until fractionation was performed.

For the cytosolic Hb fraction, the parasite pellets were resuspended in 50 μL of Milli-Q water and sonicated for 5 minutes in a water bath sonicator. Following sonication, 50 μL of 0.2M HEPES (pH 7.5) was added and the samples were centrifuged at 1500*g* for 20 minutes at 4°C. The supernatant containing the cytosolic Hb fraction was carefully transferred to new tubes and 50μL of 4% of SDS was added before the samples were incubated at 95°C for 5 min. Following heating, 50 μL of 0.3M NaCl and 50 μL of 25% (v/v) pyridine (Sigma) in 0.2M HEPES was added, the samples were vortexed and 200μl transferred to a 96-well plate. This sample contained the cytosolic Hb fraction. Absorbance at 405nm was read immediately.

The remaining pellets were resuspended with 50μL of Milli-Q water and 50μL of 4% of SDS before being sonicated for 5 minutes in the water bath sonicator and incubated at 95°C for 5 minutes in order to solubilize DV-associated Hb and free heme (FH). Following incubation, 50 μL of 0.2M HEPES, 50 μL of 0.3M NaCl and 50 μL of 25% (v/v) pyridine were added to the samples. The samples were then subsequently centrifuged at 1500*g* for 20min. 200 μL of the supernatant was transferred to the 96-well plate, corresponding to the free heme/DV-associated Hb fraction. Absorbance at 405nm was read immediately.

The remaining pellet containing the hemozoin fraction was solubilized with 50 μL of MilliQ water and 50 μL of 0.3M NaOH and then vortexed for 10 seconds. The samples were sonicated for 15 minutes before 50 μL of 0.2M HEPES, 50 μL of 0.3M HCl and 50 μL of 25% (v/v) pyridine was added. The samples were then transferred to a 96-well plate, corresponding to the hemozoin fraction.

Quantification of the heme Fe was done by comparing to a standard curve of a hematin (porcine, Sigma) solution dissolved in 0.3M NaOH. Serial dilutions were made in a 96-well plate to which the other components needed to form the heme-pyridine complex were added (0.2M HEPES pH 7.5, 4% (w/v) SDS, 0.3M NaCl, 0.3M HCl, 25% pyridine in 0.2 M HEPES pH 7.5 and Mili-Q water) and absorbance was recorded at 405nm. The amount of heme for each fraction was calculated by comparing the OD_405nm_ to the standard curve and then divided by the number of cells used in the assay. Statistics were done using a two-tailed *t* test.

### Bloated digestive vacuole assay.

The bloated DV assay was performed as described in (21, 79). 3D7 and PfPX1-KO-1 parasites were tightly synchronized by Percoll purification of late schizonts, followed 6 h later by a 5% D-sorbitol treatment (giving 0 to 6 hpi cells). Cells were put back to the incubator until the trophozoite (3D7 WT) or ring/trophozoite (PfPX1-KO line) stages after which samples of both cultures were removed to determine parasite size at the beginning of the assay. 33 mM of E64 protease inhibitor (Sigma-Aldrich) was added to 1 ml of each culture which were then incubated for 8 h, stained with 4.5 mg/ml dihydroethidium (Cayman) for 20 min at room temperature and washed once in RPMI before being imaged immediately. The DIC image was used to measure the parasite diameter and the fluorescent channel to measure the DV diameter (at least 20 cells per experiment, 3 biological replicates). Imaging and scoring of the cells in the images were done blindly to the identity of the sample.

### Growth assays.

Tight 0-5 h post invasion ring cultures were obtained by Percoll purification of schizonts, followed by invasion for 5 h in fresh RBCs and then sorbitol synchronization. 0-5 h ring staged 3D7 WT and the KO strains were seeded at 0.2% parasitemia and followed up for maturation by Giemsa staining and counting by light microscopy at various intervals till 61 h post invasion (1 and ½ cycles). After 120 h in culture, the cells were harvested and analyzed by fluorescence-activated cell sorting (FACS) on a BD FACSCanto A to calculate the parasitemia as previously described (135). Briefly, the cells were stained with SYBRGold (Invitrogen-Molecular Probe) and then fixed with 1% paraformaldehyde for 1 h. 100 000 events were acquired on the FACSCanto A using the FACSDiva software. The data were analyzed with the FlowJo software. The percentage of survival was obtained by normalizing to untreated parasites in the same experiment, which was taken as 100% survival. Uninfected red blood cells were used to determine the threshold for FITC signal. Experiments were performed with a minimum of 3 biological replicates.

For the growth assays in amino-acid deficient medium, tightly synchronized 0-5 h rings were extensively washed in PBS after which they were seeded either in complete medium or amino-acid restricted medium and processed as per above. Parasitemia was measured by FACS.

### *In vitro* 72-h susceptibility assays.

Parasite susceptibility to PI3K inhibition was measured as previously described with minor modifications (136). Briefly, sorbitol synchronized 4-to-12 h post-invasion ring stage parasites were exposed to a 16-point serial dilution of LY294002 (Sigma-Aldrich), LY303511 (Sigma-Aldrich) or DMSO in a 96-well plate at 1% hematocrit and 0.5% starting parasitaemia in 100 μl final volume. Cells were incubated for 72 h before being disrupted and the released DNA stained by adding 25 μl of 5X lysis buffer containing 0.16% saponin, 20mM Tris-HCl pH 7.5, 5mM EDTA, 1.6% Triton X-100, 5X SYBR^TM^ Gold (Invitrogen). Each assay was done in triplicate. Plates were sealed and allowed to rest at room temperature for 24 h. Relative fluorescence units (RFU) were measured with a VICTOR plate reader (PerkinElmer) at an excitation of 494 nm and emission of 530 nm. RFU were compared to untreated parasites and data plotted using GraphPad PRISM® software and IC_50_ determined using the curve-fitting algorithm *log(inhibitor) versus response – variable slope*.

### Ring survival assays.

The RSA was performed as previously described (137, 138). Briefly, cultures were sorbitol synchronized for two cycles, then 40–44h schizonts were incubated for 15 min at 37°C in incomplete media supplemented with heparin and then purified with a 35/65% discontinuous Percoll gradient. The purified schizonts were washed and cultured with fresh RBCs for 3h, after which sorbitol treatment was performed. Cultures with 0–3h rings were adjusted to 2% hematocrit and 1% parasitaemia and seeded into a 24-well plate with 1 ml complete medium per well. To the wells corresponding to RSA_0-3h_ either DHA at 70nM or 700 nM or 0.1% DMSO were added immediately and incubated for 6 h at 37°C, washed and incubated in drug free media. At 72 h from plating, thin blood smears were made from control and treated wells and parasite survival rates were measured microscopically by counting the proportion of next generation parasites with normal morphology. Survival rates were expressed as ratios of viable parasitemia in DHA-exposed and DMSO-treated controls. Parasites were estimated from 10,000 RBCs, and two separate individuals served as independent slide readers.

### Statistical analysis.

Prism 7(GraphPad) was used for all statistical analyses. Depending on the assay, one-way ANOVA or two-tailed unpaired *t* tests were performed. A minimum of 3 biological replicates were done for each experiment. A *P* value of <0.05 was considered statistically significant.
