# Supplementary figures and images for: A Phosphoinositide-Binding Protein Acts in the Trafficking Pathway of Hemoglobin in the Malaria Parasite Plasmodium falciparum
Source: mBio. 2022 Jan 18;13(1):e03239-21. doi: 10.1128/mbio.03239-21 (PMC8764524; doi:10.1128/mbio.03239-21)

# Fig. S1

A

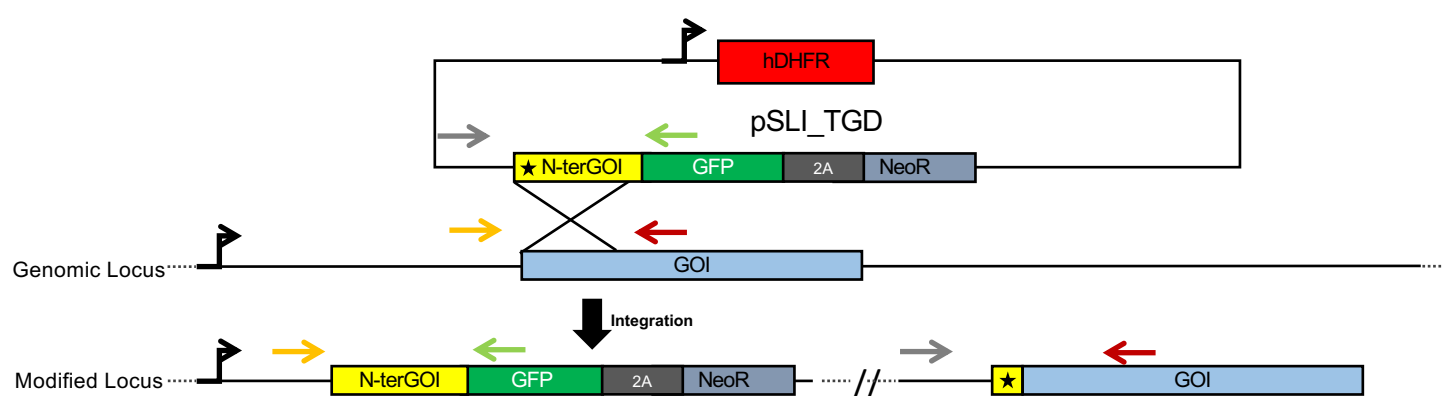

B

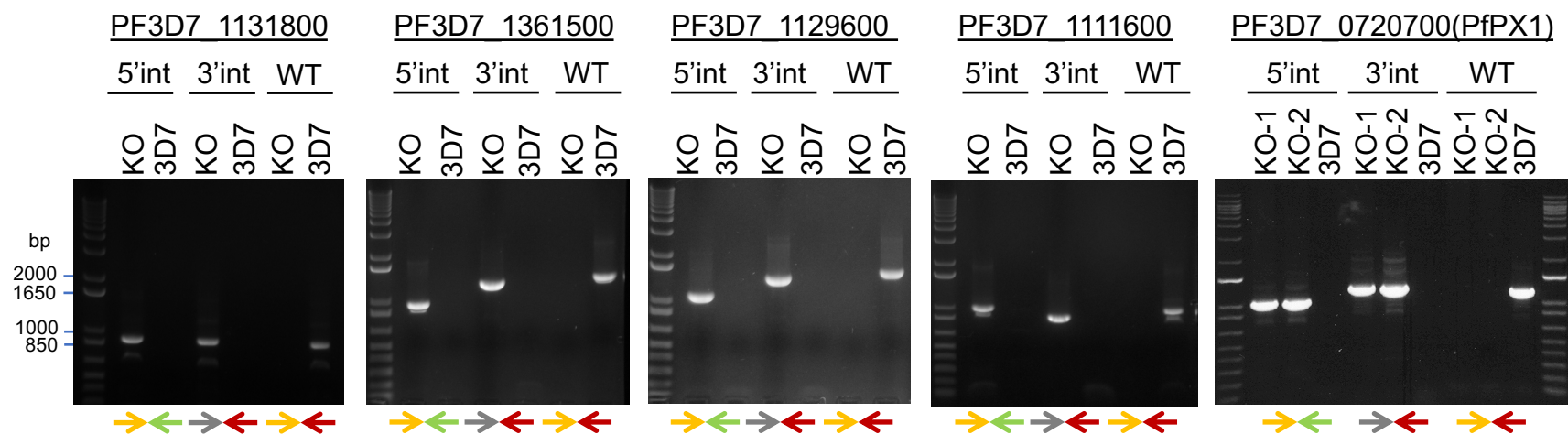

C

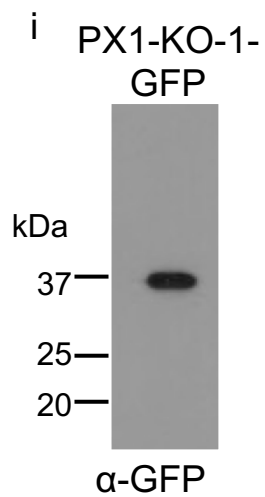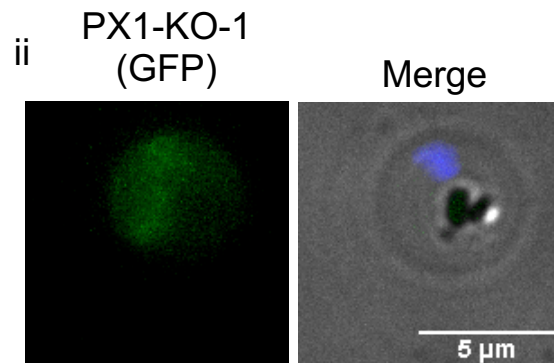

Supplement: FIG S1 [file mbio.03239-21-sf001.pdf]

Fig. S2

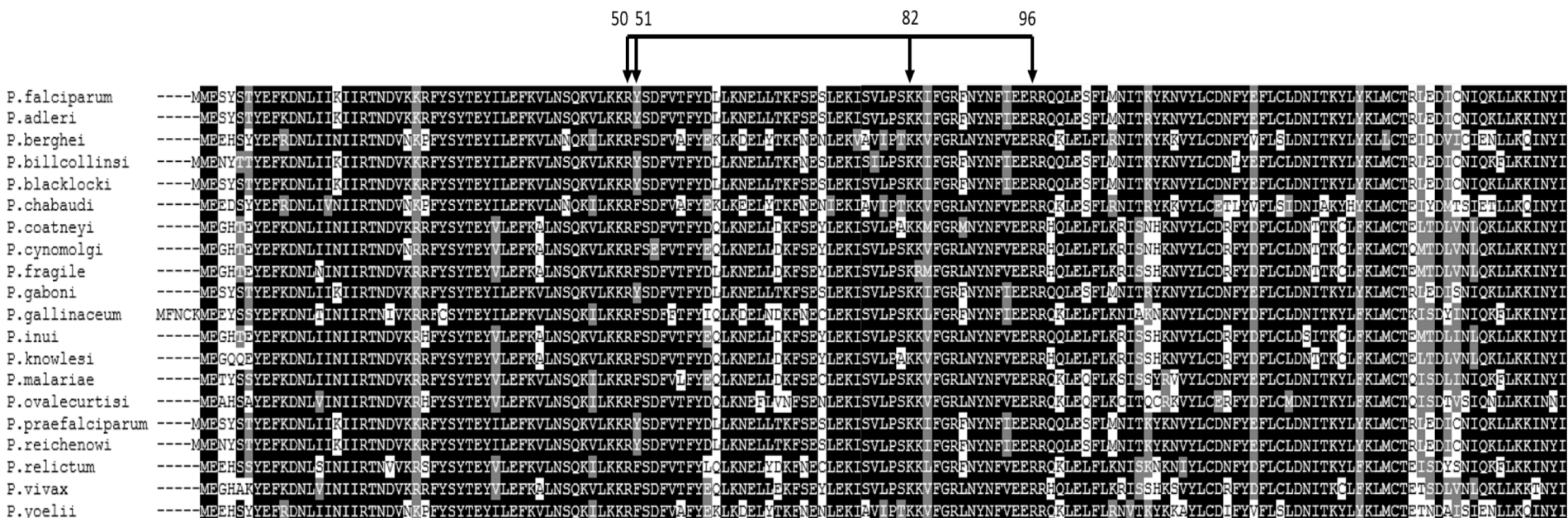

Supplement: FIG S2 [file mbio.03239-21-sf002.pdf]

Fig. S3

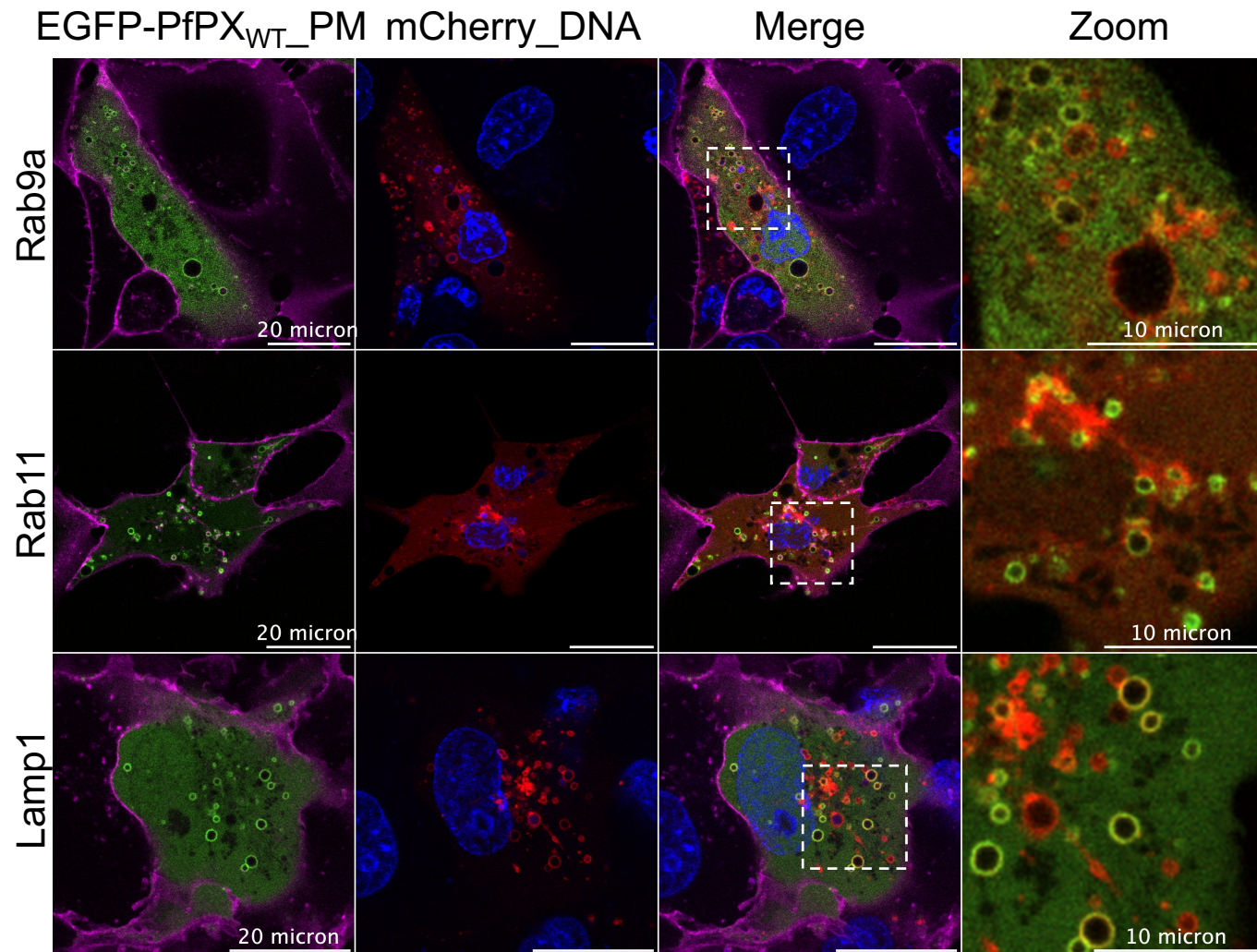

Supplement: FIG S3 [file mbio.03239-21-sf003.pdf]

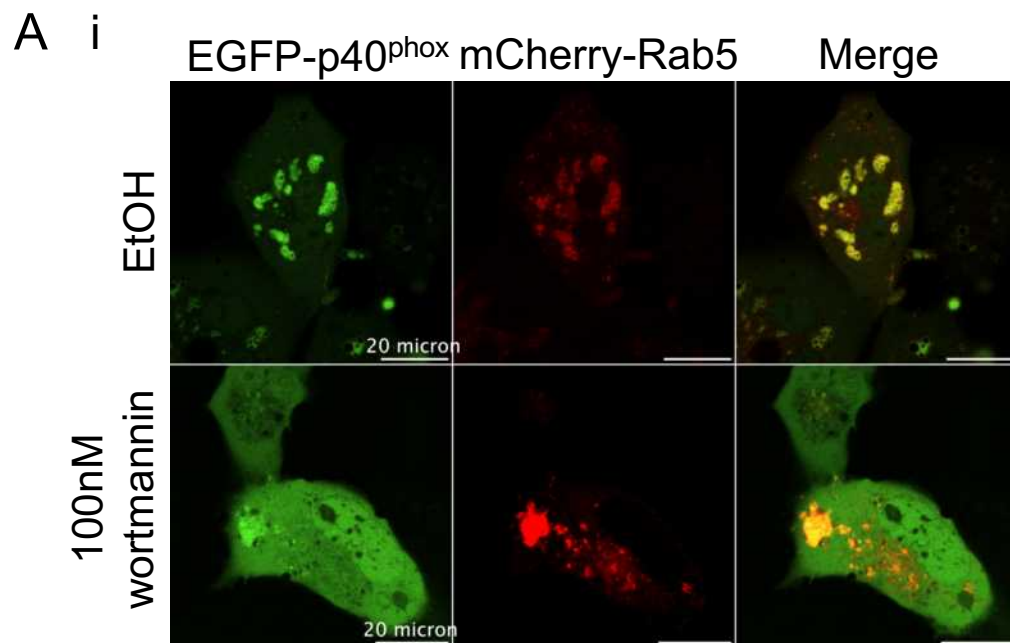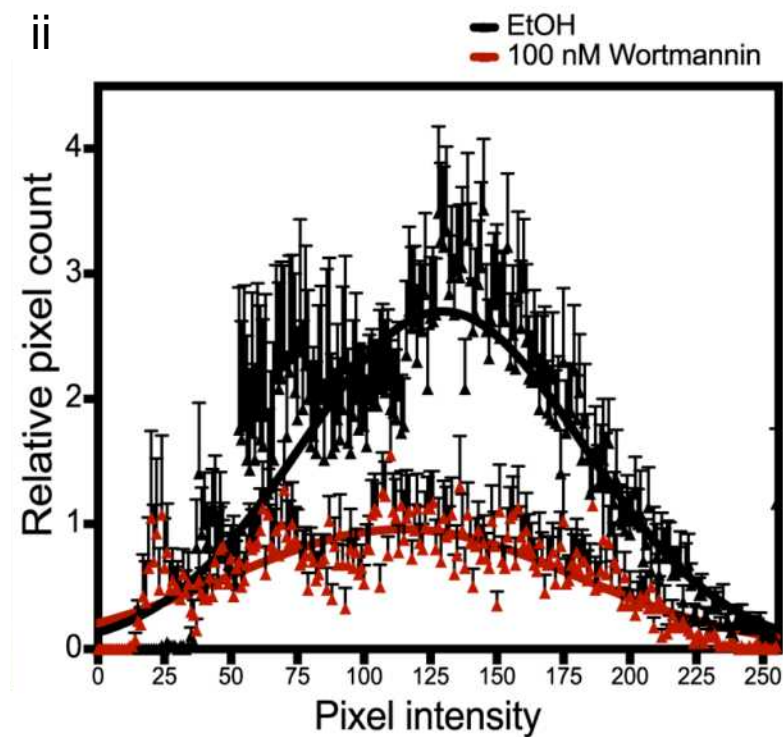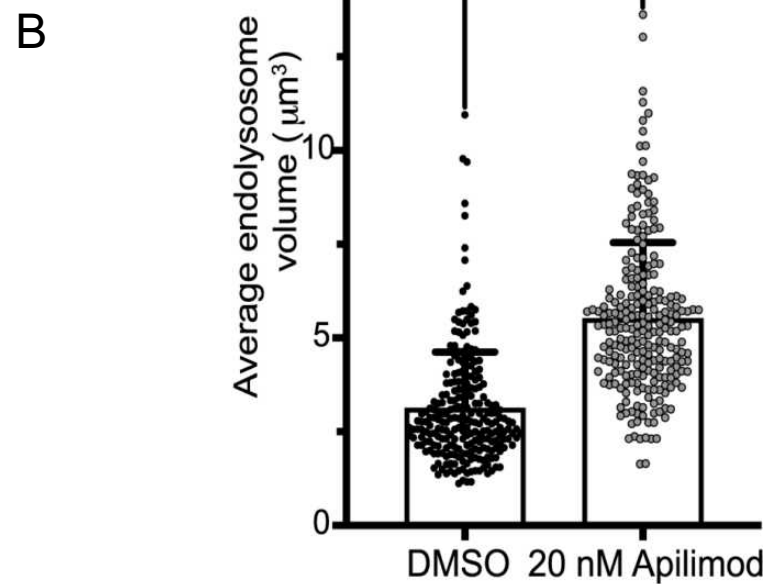

Fig. S4

Supplement: FIG S4 [file mbio.03239-21-sf004.pdf]

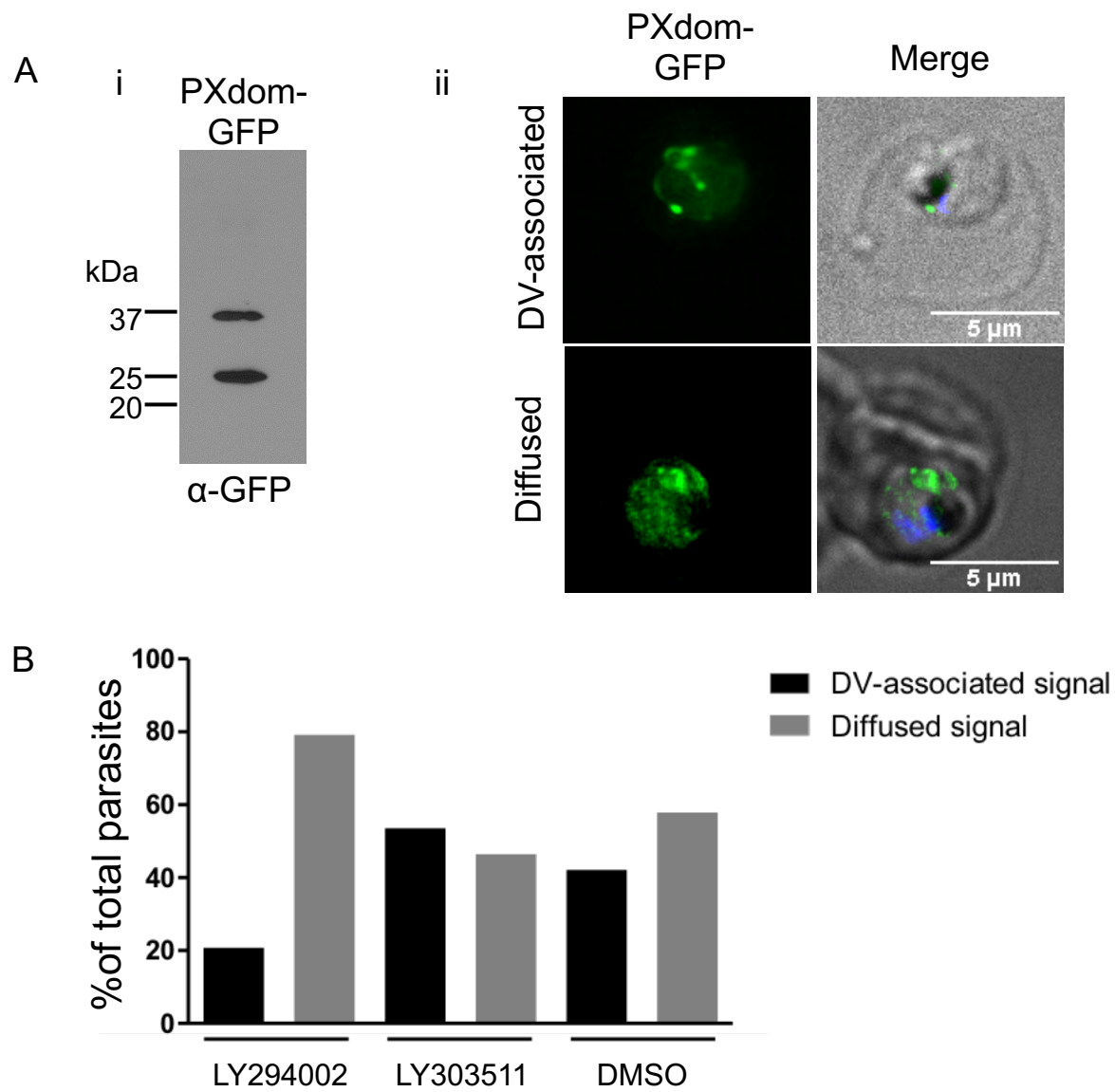

Fig. S5

Supplement: FIG S5 [file mbio.03239-21-sf005.pdf]

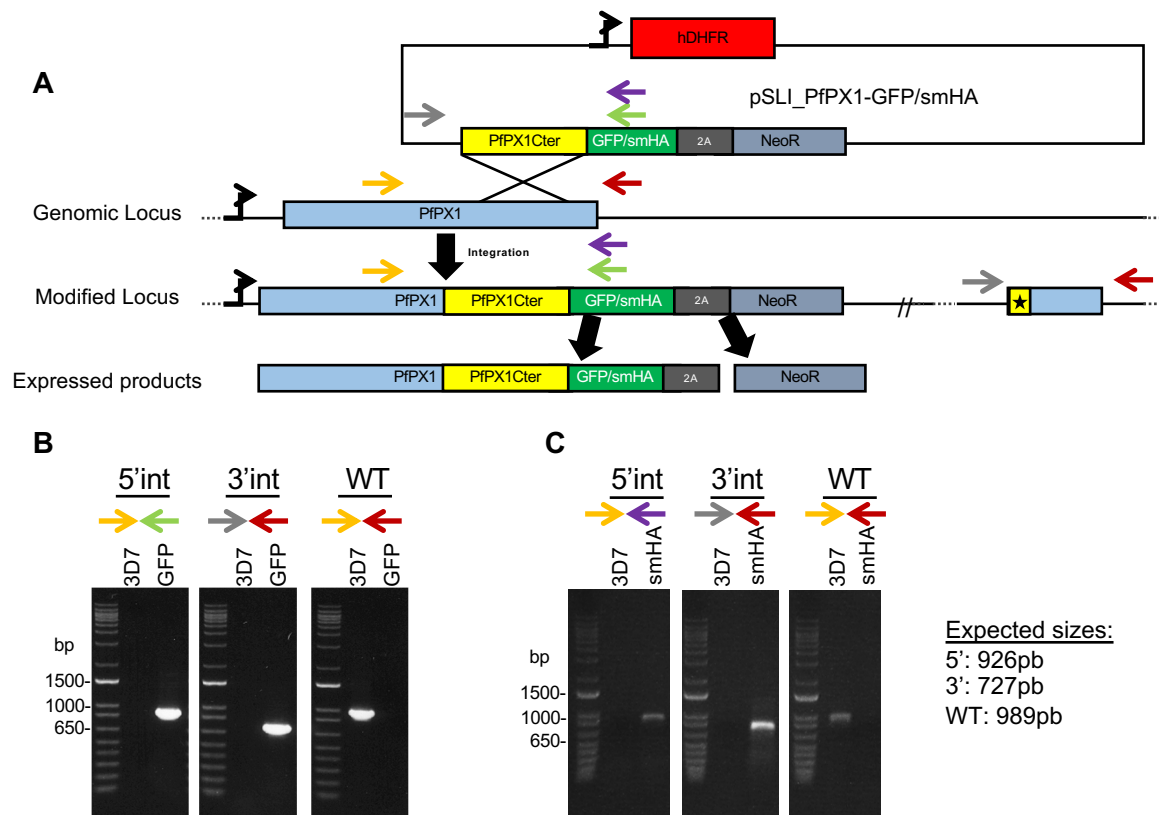

Fig. S6, Related to Fig. 4 and S7

Supplement: FIG S6 [file mbio.03239-21-sf006.pdf]

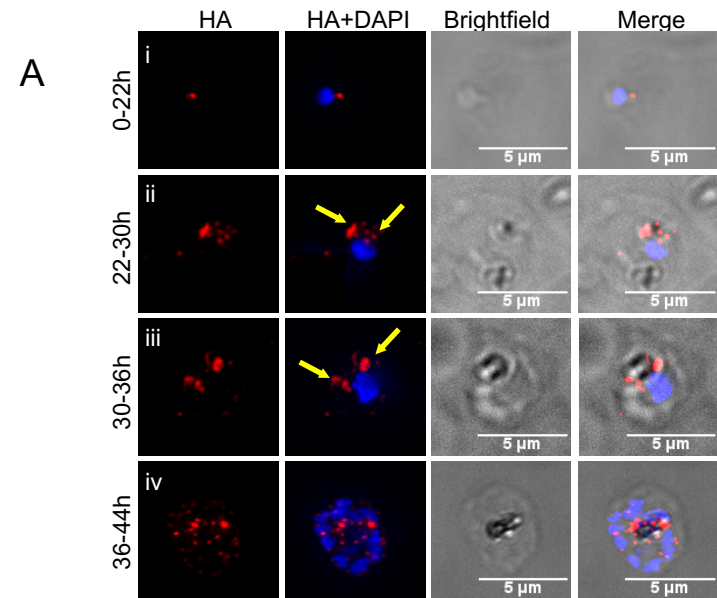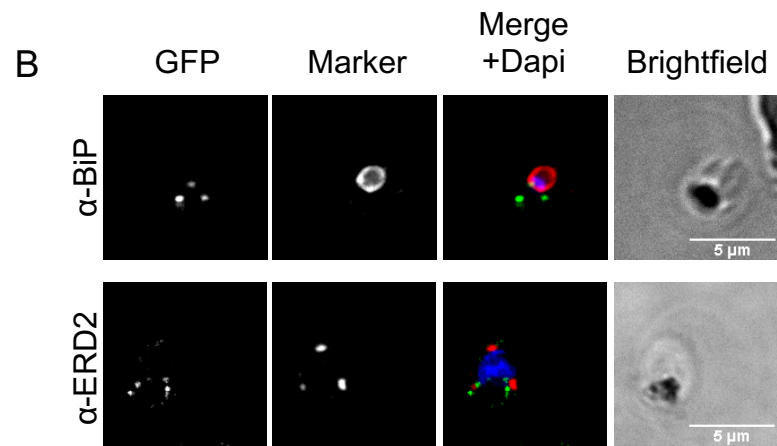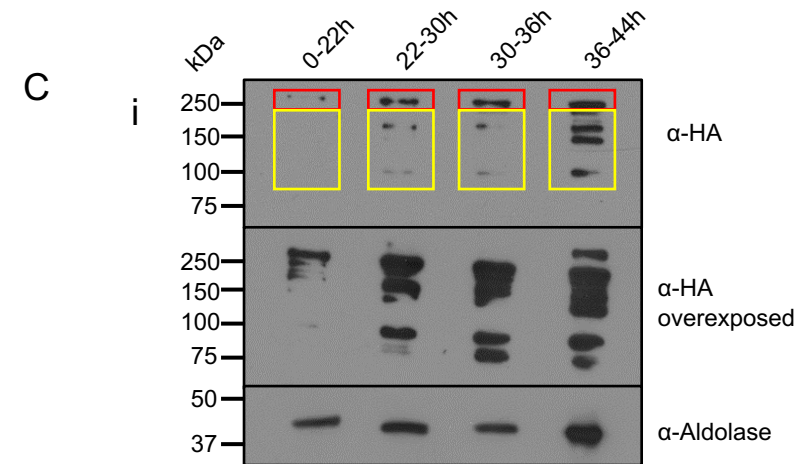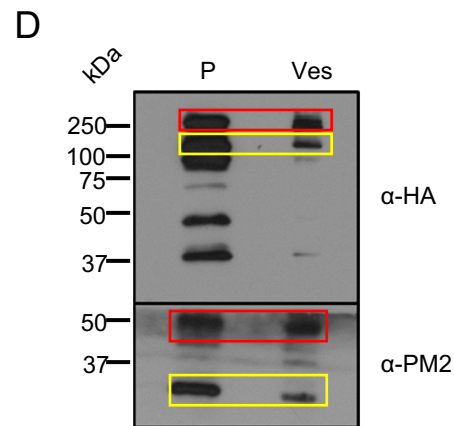

Fig S7

Supplement: FIG S7 [file mbio.03239-21-sf007.pdf]

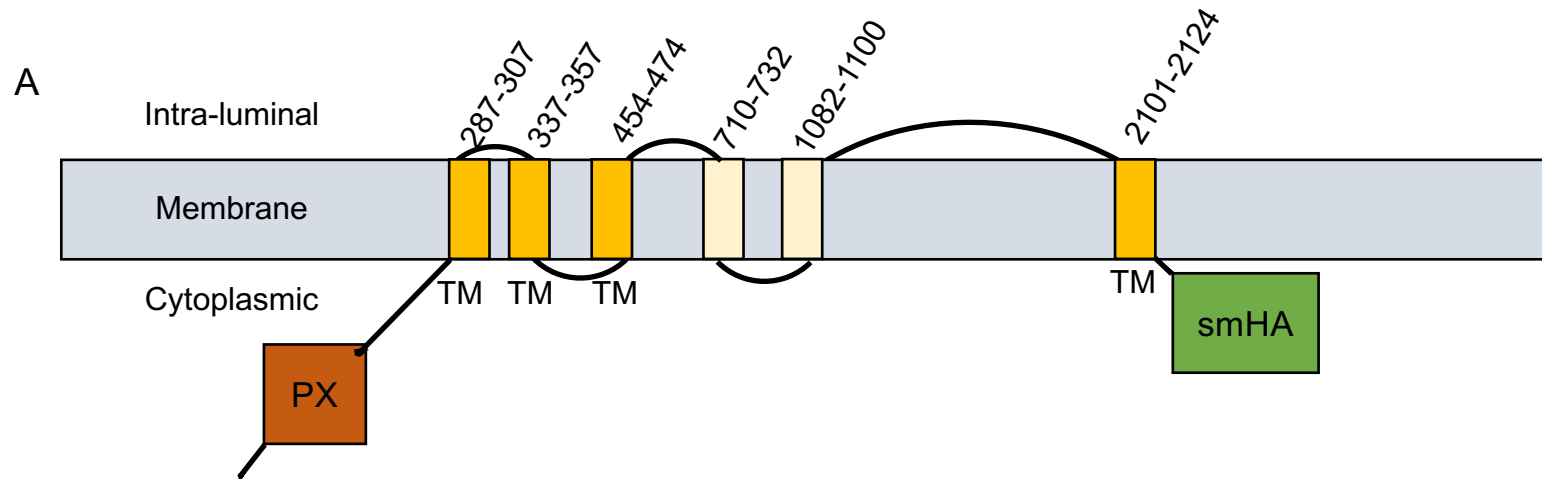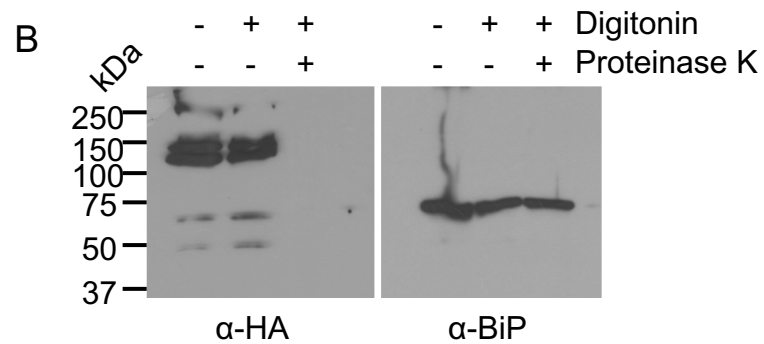

Fig S8

Supplement: FIG S8 [file mbio.03239-21-sf008.pdf]

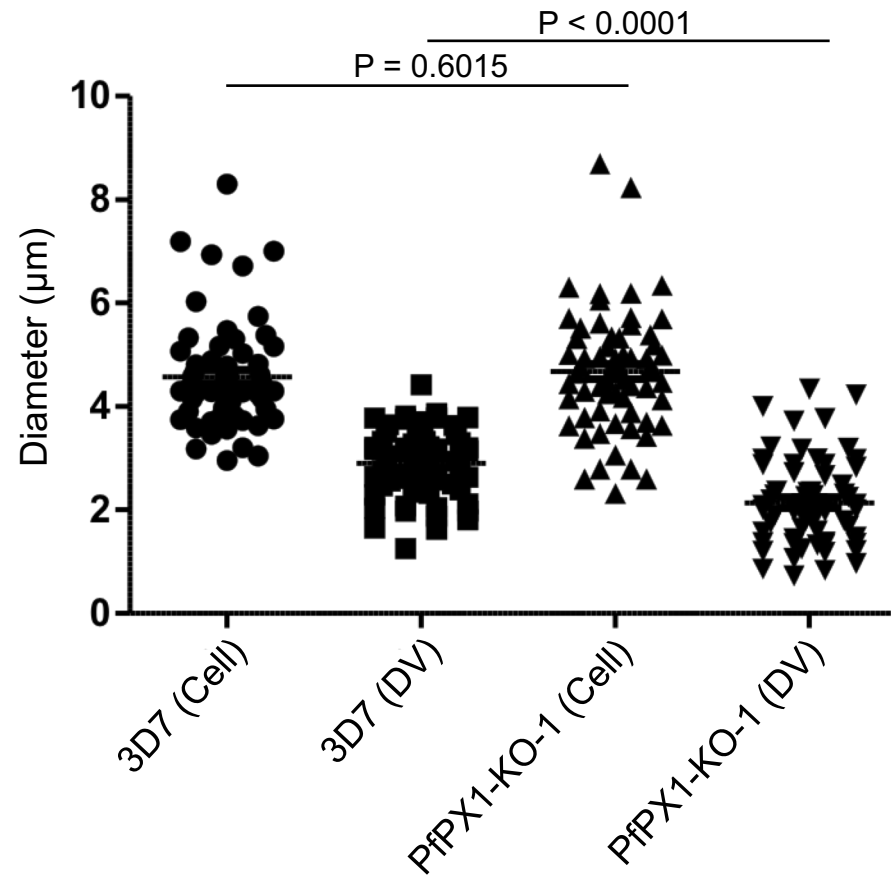

Fig. S9, Related to Fig. 6C

Supplement: FIG S9 [file mbio.03239-21-sf009.pdf]

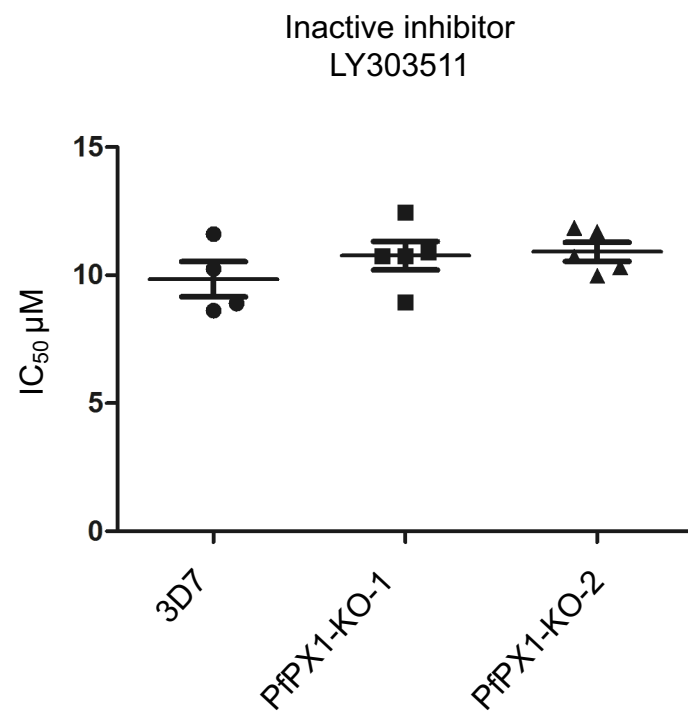

Fig. S10, Related to Fig. 7A

Supplement: FIG S10 [file mbio.03239-21-sf010.pdf]
